# Integrative review of singing and music interventions for family carers of people living with dementia

**DOI:** 10.1093/heapro/daac024

**Published:** 2022-04-13

**Authors:** Sophie Lee, Theresa Allison, Desmond O’Neill, Pattie Punch, Elizabeth Helitzer, Hilary Moss

**Affiliations:** 1 Health Research Institute, Ageing Research Centre, Irish World Academy of Music and Dance, University of Limerick, Limerick V94 T9PX, Ireland; 2 Division of Geriatrics, Department of Medicine, University of California, San Francisco, CA 94143, USA; 3 Centre for Ageing, Neurosciences and the Humanities, Trinity College Dublin, D02 PN40, Ireland; 4 Glucksman Library, University of Limerick, Limerick V94 T9PX, Ireland

**Keywords:** family carers, dementia, singing, music, integrative review

## Abstract

The majority of people living with dementia are cared for by their families. Family carers play a vital role in upholding the formal care system. Caring for a family member with dementia can be fulfilling. However, this role can have a considerable negative impact on family carers’ mental and physical health and quality of life. Several empirical research studies have recently been conducted that explore the potential benefits of music interventions for family carers of people living with dementia. Singing has been the primary musical medium employed. This article presents the first review of this literature to date. It investigates the impact of music interventions on the health and well-being of family carers of people living with dementia, and how they experience and perceive these interventions. Whittemore and Knafl’s five-stage integrative review framework was utilized: (i) problem identification; (ii) literature search; (iii) data evaluation; (iv) data analysis and synthesis; and (v) presentation of the findings. A total of 33 studies met the inclusion criteria. Analysis and synthesis resulted in three overarching themes: impact on family carers, carer perceptions of music interventions and null quantitative findings in small studies. The review found that singing and music interventions may improve family carers’ social and emotional well-being, enhance their ability to cope and care and ameliorate the caring relationship, contributing to experiences of flourishing. However, it highlighted that this area is under-researched and pointed to the need for larger, more rigorous studies.

## INTRODUCTION

The majority of people with dementia live at home where they are supported and cared for by their family (Dickinson *et al.*, 2017). As their condition progresses, people living with dementia require assistance to manage the activities of daily living and increased supervision to ensure their safety and well-being (Laver *et al.*, 2017). Family carers are a crucial determinant of positive outcomes for people living with dementia and considerably reduce the need for institutionalization (Farina *et al.*, 2017). They are central to maintaining the quality of life of people living with dementia, facilitating engagement in meaningful activities and advocating for their personhood (Teahan *et al.*, 2020b). Caring for a family member living with dementia can be hugely fulfilling (Laver *et al.*, 2017). However, this role can also be challenging and has a considerable negative impact on the family carer’s own mental and physical health and quality of life (Farina *et al.*, 2017).

There is a high incidence of carer burden among family carers of people living with dementia (Dickinson *et al.*, 2017). Alongside decline in cognitive function, responsive behaviours typically intensify as dementia progresses, making the carer’s role increasingly difficult (Hulme *et al.*, 2010). In addition, the progression of dementia can severely compromise the quality of the relationship between the person living with dementia and their family carer, augmenting feelings of loneliness for both (Clark *et al.*, 2018). Carers may experience feelings of loss due to the changes in their family member and guilt in relation to competence, burden and distress (Merrilees, 2016). Perceived strain in caring is also associated with increased risk of illness and mortality (Schulz and Beach, 1999). Family carers have a higher incidence of obesity, cardiovascular disease and hypertension, compared to controls (Vitaliano *et al.*, 1996; Mausbach *et al.*, 2007; Capistrant *et al.*, 2012). In addition, many family carers are older spouses who may be physically frail and dealing with their own conditions (Farina *et al.*, 2017).

The need to support family carers of people living with dementia has been recognized internationally (Stoltz *et al.*, 2004; Dickinson *et al.*, 2017). Alongside the importance of improving carers’ own health and well-being, family carers play a vital role in upholding the formal care system (Farina *et al.*, 2017; Watson *et al.*, 2019). According to Family Carers Ireland (Family Carers Ireland, 2018), family carers provide 6.2 million hours of unpaid care each week, while saving the State over €4 billion each year in avoided health and social care costs. In addition, research studies report multiple benefits of ageing-in-place in familiar surroundings for people living with dementia, including its ability to support optimal independence, and it is advocated for by the World Health Organization (Tamplin *et al.*, 2018; Teahan *et al.*, 2020b).

A wide variety of psychosocial interventions have been developed to promote resilience and well-being among family carers and to help them navigate their roles. These include psychoeducational interventions, multicomponent interventions, counselling and alternative interventions and activities, such as mindfulness or music (Teahan, 2020b). Systematic reviews and meta-analyses of psychosocial interventions for this population acknowledge that the heterogeneity of methodologies, study designs and contrasting intervention types impact both the synthesis and direct comparison of results. However, there is increasing evidence that psychosocial interventions can positively impact the well-being and quality of life of family carers of people living with dementia.

Music interventions are frequently utilized with the aim of improving the well-being of people living with dementia. Although family carers may be invited to participate, learn tools and skills to utilize with the person living with dementia, facilitate sessions, or suggest music that the person living with dementia enjoys listening to (Särkämö *et al.*, 2014; Garrido *et al.*, 2017; Lai and Lai, 2017), carers have only recently become the focus of music interventions. As researchers have started to recognize and explore the potential benefits that music interventions may also have for family carers, several empirical research studies have emerged, with singing as the primary musical medium employed (Unadkat *et al.*, 2017; Baker *et al.*, 2018; Clark *et al.*, 2018).

To date, no comprehensive review of this literature has been conducted. Given the accessibility and low-cost nature of group singing and other music interventions, their efficacy in improving the well-being of both non-clinical and clinical populations, including people living with dementia, and their inherent promotion of social connection, a review of existing literature would appear pertinent (Clift *et al.*, 2018; Särkämö, 2018; Fancourt and Finn, 2019). The heterogeneity of study designs and use of different validated instruments make integrative review the optimal approach to synthesize and analyse the findings to date. We therefore designed an integrative review that aims to answer the following two research questions:

1. How do music interventions impact the health and well-being of family carers of people living with dementia?
2. How are music interventions perceived among family carers of people living with dementia? Specifically, in relation to the suitability of music interventions, and the practicalities of engagement, including cost.

## METHODOLOGY AND METHODS

Whittemore and Knafl’s (Whittemore and Knafl, 2005) five-stage integrative review framework was employed: (i) problem identification; (ii) literature search; (iii) data evaluation; (iv) data analysis and synthesis; and (v) presentation of the findings. Six researchers were involved in carrying out this review.

### Problem identification

Studies were required to meet the following inclusion criteria: (i) peer-reviewed, (ii) written in the English language and (iii) captured the impact of engaging in a music intervention on aspect(s) of family carers’ own health and well-being and/or their perceptions of the music intervention. Studies were excluded if they were (i) not empirical primary research studies, (ii) background articles, discussion or opinion pieces, or reviews, (iii) studies where family carers participated in a music intervention but data about their experiences and/or perceptions were not collected and (iv) studies where it was not possible to extract data specific to family carers.

### Literature search

A comprehensive systematic search of the literature was conducted to identify the maximum number of eligible primary sources using four search strategies (Jadad *et al.*, 1998; Conn *et al.*, 2003; Whittemore and Knafl, 2005).

1. Relevant electronic databases were identified. A search strategy was developed and adjusted to fit each electronic database. The search strategies are included in Supplementary material A. The following electronic databases were searched 23–25 November 2020: Academic search complete/EBSCO; AMED/EBSCO; Cinahl/EBSCO; Cochrane Controlled register of Trials; medline/Ovid; psycINFO/EBSCO; PubMed; Sage; Science Direct; SCOPUS; and Web of Science. The search was limited to English language studies. Initial search results were crosschecked for consistency during research meetings. The references of the studies identified were imported into EndNote, deduplicated and uploaded to Covidence for review. The first and final authors independently completed the initial title and abstract screening, looking for relevance based on the inclusion and exclusion criteria. A meeting was held with the research team to mediate and resolve any disagreements. The first and final authors then independently reviewed the full texts of the included studies. A second meeting of the research team was held and a final decision made on which studies to include.
2. SCOPUS and ProQuest were searched for peer-reviewed conference papers and proceedings, dissertations and theses. The systematic search of these databases was conducted on 15 December 2020 and followed the steps outlined above.
3. A hand search of non-ISI journals relating to music therapy and/or music and health including *Music Therapy Perspectives, Music and Medicine*, and *Voices: A World Forum for Music Therapy* was conducted 8–12 February 2021. Potential studies for inclusion were identified, shared with the research team and reviewed at a research meeting, where the final decision was made.
4. An ancestry search of reference lists of eligible studies and review articles was conducted. Potential studies for inclusion were identified, sourced, shared with the research team and reviewed at a research meeting, where the final decision was made.

In any instance where a potentially relevant full-text could not be located, or clarification was necessary, the author was contacted. Results of the literature search can be seen in Supplementary Figure S1.

### Data evaluation

The quality and methodological rigour of each included study was evaluated using the Mixed Methods Appraisal Tool (MMAT) Version 2018, as it caters for multiple methodological designs (Hong *et al.*, 2018). The studies were each reviewed by the first author in accordance with the relevant appraisal checklist and a selection independently reviewed by the final author. In any instances when the first author questioned an aspect of their evaluation, or the appraisals of the first and final authors differed, opinions were sought from the other members of the research team and a consensus reached. As advocated in other integrative reviews, no studies were excluded on the grounds of quality appraisal (Noonan *et al.*, 2017; McCaffrey *et al.*, 2020). However, the findings of the appraisal process highlight methodological issues in some studies, which were considered when reporting overall study findings.

The results of the MMAT are presented in Supplementary Material C Tables S2a–2D. Results for the qualitative studies indicate that they are generally of high quality, although, insufficient reporting of the analysis process was noted in four cases (Supplementary Table 2a). The quality of the RCT (Särkämö *et al.*, 2014) was similarly perceived to be high, as the issue with baseline group differences (Q2) was controlled for statistically (Supplementary Table 2b). Results for the quantitative and mixed methods studies indicate that they are typically of moderate quality. In the quantitative studies, this appears to be attributable to the lack of representative samples of target populations (Q1), and that confounders were not accounted for in design and analysis (Q4) (Supplementary Table 2c). In the mixed methods studies, lack of rationale given for using mixed methods (Q1) and issues with quality criteria (Q5) negatively impacted their ratings (Supplementary Table 2d).

### Data analysis and synthesis

Analysis and synthesis of the included studies were facilitated by the creation of a review matrix and employment of thematic analysis (Braun and Clark, 2006, 2019; Garrard, 2017; Dwyer, 2020). The studies were read multiple times by the first and final authors. Categories of information to extract were proposed to the research team and agreed upon. This information was presented in a review matrix (Dwyer, 2020), to facilitate the systematic comparison, analysis and synthesis of primary data sources recommended by Whittemore and Knafl (Whittemore and Knafl, 2005).

Next, the qualitative and quantitative data were analysed following the recursive six-stage process of thematic analysis, as outlined by Braun and Clark (Braun and Clark, 2006, 2019). Although thematic analysis is most commonly used in qualitative data analysis, the approach can also be used to ‘identify and organize the main, recurrent, or most important themes or concepts across multiple sources of literature’ [(Dwyer, 2020), p. 66; Popay *et al.*, 2006]. The first and final authors independently assessed the review matrix and engaged in an iterative process of coding, noting themes, patterns and relationships (Braun and Clarke, 2006, 2019). They each organized their codes into potential themes and then met to review, compare and discuss them (Braun and Clarke, 2006, 2019). They considered whether the themes needed to be combined, refined, separated or discarded, as well as the validity of individual themes in relation to the dataset as a whole (Braun and Clarke, 2006, 2019). The second and third authors mediated this process. The themes were subsequently refined and re-named until synthesis of the final themes was achieved (Braun and Clarke, 2006, 2019).

## RESULTS

### Descriptive results

A total of 33 studies met the inclusion criteria for this review. The results of two of the included studies were reported across two publications, meaning that 35 publications were included in the review. They comprised 30 journal articles, 2 book chapters, 2 doctoral theses and 1 master’s thesis. The studies’ countries of origin include Australia (*n* = 7), Canada (*n* = 1), England (*n* = 5), England and Wales (*n* = 1), England and Japan (*n* = 1), Finland (*n* = 1), Ireland (*n* = 1), Israel (*n* = 1), Italy (*n* = 1), Scotland (*n* = 1), Spain (*n* = 2) and USA (*n* = 12). The date range of the studies spans 1993–2020. Of the studies included, 5 were published during the 1990s (14.29%), 2 from 2000 to 2010 (5.71%) and 28 between 2011 and 2020 (80%).

The studies had diverse methodological designs. Fourteen studies were qualitative. Data collection methods varied across these studies and included semi-structured interviews, focus groups, observations, structured observations, field notes, journal logs and open-ended questionnaires. Two of the qualitative studies were part of the same project (Harris and Caporella, 2014, 2019). One study was a randomized controlled trial and used both quantitative and qualitative methods of data collection (Särkämö *et al.*, 2013, 2014). Fifteen studies employed a quasi-experimentalrepeated-measures design. Ten of these studies used mixed methods, most commonly capturing qualitative data in the form of post-intervention interviews. One of these studies reported the results across two papers (Clark *et al.*, 2018; Tamplin *et al.*, 2018). Five used only quantitative measures. The quantitative psychological measurement tools for health and well-being utilized are included in Supplementary material D. One additional study used a quantitative post-test-only design (Klein and Silverman, 2012). The final two studies used other mixed methods designs (Clair, 2002; Zeilig *et al.*, 2019).

A total of 342 family carers participated in the studies. The smallest sample size of family carers was *n *=* *2 (Hanser and Clair, 1995; Gardner, 1999; Dassa *et al.*, 2020) and the largest was *n *=* *59 (Särkämö *et al.*, 2013, 2014). Gender was reported in 26 studies (28 publications). Of family carers, 180 were women (52.63%); 86 were men (25.14%); and gender was not reported for the remaining 76 participants (22.22%). The relationship between each family carer and their care recipient was reported in 28 studies (29 publications). Two hundred family carers were spouses/partners of people living with dementia (58.48%); 58 were children of people living with dementia (17.00%); 7 were siblings of people living with dementia (2.05%); 15 were other named family carers (4.39%); and the relationship between 62 caring dyads was not reported (18.13%). Age was reported in 22 studies (23 publications). The youngest family carer reported was 32 years old and the oldest was 90. Ethnicity was only reported in eight studies. The ethnicity of 79 family carers was reported (23.10%) and the ethnicity of 263 family carers was not reported (76.90%).

The music interventions employed included music therapy (*n* = 11), therapeutic songwriting (*n* = 5), group singing (*n* = 9) and other (*n* = 8). Group music interventions were designed for family carers to attend alone (*n* = 3) or with their family member with dementia (*n* = 30). Four music interventions were designed for individual spousal dyads, and six for individual familial dyads. The music interventions took place in community settings (51.52%) in 17 studies, participants’ homes (21.21%) in 7 studies, residential care settings (18.18%) in 6 studies, in participants’ homes and a residential care setting (3.03%) in 1 study, and in community and residential settings (6.06%) in 2 studies. The review matrix and details of the music interventions are included in Supplementary materials E and F.

### Results of thematic analysis and synthesis

Analysis and synthesis resulted in three overarching themes: impact on family carers, carer perceptions of music interventions and null quantitative findings in small studies (Braun and Clarke, 2006, 2019; Dwyer, 2020). Supplementary material G contains figures S2 and S3 that illustrate the frequency of subthemes within overarching themes.

#### Theme 1: impact on family carers

##### Amelioration of caring relationship

Nineteen studies found that participating in the music intervention enhanced the quality of the relationship between the family carer and the person that they cared for. Studies captured how participating in a music intervention together facilitated positive identity construction, increasing awareness of the person living with dementia’s identity outside of their diagnosis (Dupuis and Pedlar, 1995; Gardner, 1999; Davidson and Almeida, 2014; Unadkat *et al.*, 2017). They noted the ability of the music interventions to ‘bring back’ people living with dementia to their family carers [(Clair and Ebberts, 1997), p. 158; Melhuish *et al.*, 2019; Dassa *et al.*, 2020] and to partially restore lost or diminished aspects of their personalities (Gardner, 1999; Shibazaki and Marshall, 2017). They captured how musical reminiscence allowed them to access their relationship before dementia (Baker *et al.*, 2012; Davidson and Almeida, 2014). It was also reported that meeting in a space where their caring roles could be forgotten facilitated reconnection and feelings of equity (Garabedian and Kelly, 2020; Lee *et al.*, 2020).

Several studies reported that the music intervention strengthened reciprocity between the dyad (Dupuis and Pedlar, 1995; Clair and Ebberts, 1997; Baker *et al.*, 2012; Macgregor, 2016; Unadkat *et al.*, 2017; Melhuish *et al.*, 2019). Reciprocity was a major focus of Macgregor’s study (Macgregor, 2016). They found that family carers were longing for emotional reciprocity and that they were able to develop non-verbal methods of communication and elicit emotional reciprocity through music (Macgregor, 2016). Positive touch was similarly highlighted as a meaningful method of non-verbal communication in Clair and Ebberts’ study (Clair and Ebberts, 1997). They reported that, although family carers and care recipients both initiated and received touch from one another during the music therapy sessions, family carers had low response rates to touch (Clair and Ebberts, 1997). These findings suggest that, although music can facilitate emotional reciprocity, family carers may initially need to adjust their expectations and become more perceptive to non-verbal communication.

Several studies also found that the music interventions enhanced the quality of the time spent together, facilitated meaningful interactions, stimulated conversation and gave the dyads an opportunity to make new memories together (Dupuis and Pedlar, 1995; Hanser and Clair, 1995; Clair, 2002; Baker *et al.*, 2012; Osman *et al.*, 2016; Shibazaki and Marshall, 2017; Clark *et al.*, 2018). Clair (Clair, 2002) reported a statistically significant improvement in engagement scores following implementation of the individualized music protocols by the family carers. However, the low quality of this study (MMAT) should be noted.

##### Improved social well-being

Sixteen studies reported that participation in the music interventions increased family carers’ feelings of social connection and support. Each of these studies found that family carers valued the opportunity to meet other carers who could relate to what they were experiencing (Baker and Yeates, 2018; Clark *et al.*, 2018). Dupuis and Pedlar [(Dupuis and Pedlar, 1995), p. 191] reported the formation of an ‘empowering supportive network’ for family carers.

Feelings of connection and empathetic support were reported in studies where the music interventions focussed on family carers’ experiences of caring, such as therapeutic songwriting in Baker *et al.* (Baker *et al.*, 2018), and studies where the music intervention did not, such as the ‘Singing Together’ group in Camic *et al.* (Camic *et al.*, 2013). Baker *et al.* (Baker *et al.*, 2018) found that therapeutic songwriting enabled participants to share the whole carer experience, including their emotional journeys. It captured the value of hearing other carers’ stories and struggles, providing context for personal experiences. Clark *et al.* (Clark *et al.*, 2018) also noted that the collaborative nature of songwriting necessitated interaction and fostered connection between the carers. In contrast, Camic *et al.* (Camic *et al.*, 2013) found that the participants valued meeting other family carers in an environment where the focus was not on dementia. The accessibility of dementia-inclusive interventions was seen to support social inclusion, with family carers reported to enjoy attending a social activity with their care recipient where they did not have to worry about how they might act or respond (Dupuis and Pedlar, 1995; Camic *et al.*, 2013; Osman *et al.*, 2016; Lee *et al.*, 2020).

Reduced feelings of isolation and loneliness, and increased feelings of belonging and solidarity were reported across the studies (Dupuis and Pedlar, 1995; Harris and Caporella, 2014). Harris and Caporella (Harris and Caporella, 2019) and Zeilig *et al.* (Zeilig *et al.*, 2019) both referenced a sense of community that developed between their participants. In addition to caring, these groups bonded over musical interests and shared musical goals (Harris and Caporella, 2014; Unadkat *et al.*, 2017; Baker and Yeates, 2018; Clark *et al.*, 2018). Studies reported that strong relationships and friendships were formed between carers and with other group members (Clair *et al.*, 1993; Harris and Caporella, 2014, 2019; Unadkat *et al.*, 2017).

##### Enhanced emotional well-being

Fifteen studies reported that the music intervention positively affected one or more aspects of the family carers’ emotional well-being. Nine studies described how participation in the music intervention boosted the moods of the family carers. Davidson and Almeida (Davidson and Almeida, 2014) reported statistically significant increases in mood. Hanser *et al.* (Hanser *et al.*, 2011) similarly reported statistically significant increases in perceived levels of relaxation, comfort and happiness. In some studies, improved mood was attributed to the experience of making music (Osman *et al.*, 2016; Unadkat *et al.*, 2017; Clark *et al.*, 2018). In others, family carers spoke about the positive impact that seeing their care recipients engaged, happy and responsive had on their own moods (Baker *et al.*, 2012; Davidson and Almeida, 2014; Mittelman and Papayannopoulou, 2018; Clark *et al.*, 2020; Garabedian and Kelly, 2020; Lee *et al.*, 2020). Eight studies found the music interventions to be relaxing for family carers, or a distraction from stress (Hanser and Clair, 1995; Brotons and Marti, 2003; Baker *et al.*, 2012; Klein and Silverman, 2012; Camic *et al.*, 2013; Davidson and Almeida, 2014; Garabedian and Kelly, 2020; Lee *et al.*, 2020). Three studies also noted that the music interventions facilitated expression of emotions, with Baker and Yeates (Baker and Yeates, 2018) describing the experience as cathartic (Brotons and Marti, 2003; Baker *et al.*, 2018).

Two studies reported negative responses in relation to emotional well-being, in addition to positive ones (Davidson and Almeida, 2014; Garabedian and Kelly, 2020). Garabedian and Kelly (Garabedian and Kelly, 2020) found that some family carers experienced tension due to feeling compelled to focus on their care recipients’ responses throughout the music intervention. Of the data collected by Davidson and Almeida (Davidson and Almeida, 2014), 12% described instances where the music intervention failed to improve the mood or relax a family carer.

##### Benefits for coping and caring

Twenty-one studies reported that participating in the music intervention helped family carers to cope with their caring role. Dassa *et al.* (Dassa *et al.*, 2020) found that music became an additional caring tool for the family carers. The carers used it to stimulate, motivate, calm and/or improve the mood of their care recipient (Dassa *et al.*, 2020). Similarly, the family carers in other studies are planning to, or have continued to, use music with their care recipients (Clair, 2002; Camic *et al.*, 2013; Dowlen, 2018).

The music interventions gave family carers an opportunity to contribute to their care recipient’s well-being (Dupuis and Pedlar, 1995; Lee *et al.*, 2020). Gardner (Gardner, 1999) described how music enabled a family carer to play an active role in soothing and comforting their care recipient, meeting their needs in-the-moment and reducing the carer’s feelings of helplessness. Carer empowerment and increased control were similarly expressed in studies by Baker *et al.* (Baker *et al.*, 2018) and Melhuish et al. (Melhuish *et al.*, 2019). This helped to alleviate feelings of guilt of family carers who had institutionalized their care recipients and the challenges of visiting (Dupuis and Pedlar, 1995; Clair and Ebberts, 1997; Shibazaki and Marshall, 2017; Garabedian and Kelly, 2020).

Studies also reported that the music interventions fostered feelings of inner strength, personal growth, increased resilience, social confidence and self-esteem amongst family carers (Hanser and Clair, 1995; Baker *et al.*, 2018; Baker and Yeates, 2018; Clark *et al.*, 2018). Särkämö *et al.* (Särkämö *et al.*, 2013) described how they provided experiences of success for the carer dyads. Both García-Valverde *et al.*(García-Valverde *et al.*, 2020) and Mittelman and Papayannopoulou (Mittelman and Papayannopoulou, 2018) reported a statistically significant increase in self-esteem across their interventions. Baker *et al.* (Baker *et al.*, 2018) and Unadkat *et al.* (Unadkat *et al.*, 2017) found that participating in the music interventions challenged assumptions that asking for help was a sign of not being able to cope. Participation helped to shift their self-expectations, enhancing their ability to care (Unadkat *et al.*, 2017; Baker *et al.*, 2018). Empathetic support and shared experiences offered participants new perspectives and reflections on their role as a carer (Baker *et al.*, 2018; Baker and Yeates, 2018). Baker *et al.* (Baker *et al.*, 2018) reported that the song the group composed became a personal resource that they could draw on to support their ability to cope with the challenges of caring. Dupuis and Pedlar (Dupuis and Pedlar, 1995) and Osman *et al.* (Osman *et al.*, 2016) found that participating in a music intervention also helped family carers to be more accepting of their care recipient’s diagnosis and their situation. Participation also encouraged self-care (Mittelman and Papayannopoulou, 2018), releasing carers from their responsibilities and allowing them to take some time for themselves (Baker *et al.*, 2012; Unadkat *et al.*, 2017; Dassa *et al.*, 2020). Raglio *et al.* (Raglio *et al.*, 2016) and Särkämö *et al.* (Särkämö *et al.*, 2014) both reported statistically significant decreases in carer burden post-intervention. However, participating in a music intervention did not appear to increase satisfaction with the caring role (Baker *et al.*, 2012).

#### Theme 2: carer perceptions of music interventions

##### Accessibility

The accessibility of music, particularly group singing, for people living with dementia and their family carers, was highlighted in eight studies (Osman *et al.*, 2016; Unadkat *et al.*, 2017). Participants reported an innate ability and desire to sing (Unadkat *et al.*, 2017). Clark *et al.* (Clark *et al.*, 2018) found that the levelling, non-judgmental environment of the therapeutic singing group supported participants to continue singing or to ignite a new passion (Clark *et al.*, 2018). Suitable facilitation, musical content, setting and structure were seen as vital to accessibility (Camic *et al.*, 2013; Unadkat *et al.*, 2017; Clark *et al.*, 2018; Mittelman and Papayannopoulou, 2018; Tamplin *et al.*, 2018; Clark *et al.*, 2020; Lee *et al.*, 2020). Baker *et al.* (Baker *et al.*, 2012) also found that past experiences together involving music contributed to the effectiveness and suitability of the intervention for carer dyads.

However, barriers to engagement were also reported. Family carers identified challenges of therapeutic songwriting for people living with dementia, such as difficulty maintaining focus (Clark *et al.*, 2020). Similarly, although the improvisatory nature of the co-creative intervention encouraged self-sufficiency, freedom and agency, some participants reported that the lack of distinct boundaries and a clear direction made them feel uncomfortable and vulnerable (Zeilig *et al.*, 2019). Hanser *et al.* (Hanser *et al.*, 2011) and Macgregor (MacGregor, 2016) also reported challenges encountered by family members when leading sessions themselves and the need for increased support from music therapists. Lastly, due to a perceived lack of musical ability, some participants were initially apprehensive about what they could contribute (Camic *et al.*, 2013; Baker and Yeates, 2018). However, following the interventions, these participants reported being pleasantly surprised with how accessible they found it and the quality of what they were able to achieve (Camic *et al.*, 2013; Baker and Yeates, 2018).

##### Enjoyable

Thirty studies reported that the family carers found the music intervention enjoyable. High satisfaction ratings were found in studies by Camic *et al.* (Camic *et al.*, 2013) and Clair and Ebberts (Clair and Ebberts, 1997). Clair and Ebberts (Clair and Ebberts, 1997) noted that carers gave significantly higher satisfaction ratings for visits with music therapy. Enjoyment was attributed to different aspects of the music interventions. Several studies found that family carers valued the cognitive stimulation that the music interventions provided (Clair *et al.*, 1993; Unadkat *et al.*, 2017; Tamplin *et al.*, 2018; Clark *et al.*, 2020). They were also reported to enjoy the challenge of learning new things and developing new or existing skills (Clair *et al.*, 1993; Hanser and Clair, 1995; Clark *et al.*, 2018; Mittelman and Papayannopoulou, 2018). The opportunity to be creative and the sense of achievement afforded by having end goals, such as a performance or song composition, were similarly cited as positive aspects of participation (Harris and Caporella, 2014; Baker *et al.*, 2018; Baker and Yeates, 2018; Clark *et al.*, 2020). The enjoyable experience of singing with others was also reported, along with the escapism it can provide (Camic *et al.*, 2013).

Seven studies included family carers perceptions of the practical elements that contributed to their enjoyment (Camic *et al.*, 2013; Unadkat *et al.*, 2017; Clark *et al.*, 2018; Mittelman and Papayannopoulou, 2018; Tamplin *et al.*, 2018; Clark *et al.*, 2020; Lee *et al.*, 2020). These included supportive, inclusive facilitation, participant involvement in choice of musical content, suitable setting and appropriate session time, length and structure. The desire for the intervention to continue, reported in several studies, is similarly demonstrative of participant enjoyment (Clair *et al.*, 1993; Clark *et al.*, 2018; Tamplin *et al.*, 2018).

Baker *et al.* (Baker *et al.*, 2018) and Clark *et al.* (Clark *et al.*, 2020) found that the therapeutic songwriting intervention addressed an important need for family carers not necessarily met by other support groups. Rather than focussing on information sharing and addressing day-to-day challenges, this intervention allowed the participants to express their thoughts and feelings around being a carer (Baker *et al.*, 2018). It allowed the participants to additionally focus on the positives of caring, which are typically overshadowed by topics, such as stress or burden (Baker *et al.*, 2018). It also placed an emphasis on collaboration, as opposed to individual contexts, which fostered unity within the group (Baker and Yeates, 2018).

#### Theme 3: null quantitative findings in small studies

Seventeen studies employed quantitative psychological measures as part of a quasi-experimentalrepeated-measures (*n* = 16) or a randomized controlled trial (*n* = 1) design. The heterogeneity of study design and use of different validated instruments made it impossible to conduct a meta-analysis. Fifty psychological measures (34 different psychological tools) were used across the studies to investigate the impact of participation in a music intervention on an aspect or multiple aspects of the family carers’ health and well-being. Statistical significance was only found for a measure, or subscales of a measure, in thirteen cases, resulting in a low level of quantitative evidence.

Change in anxiety was measured in four studies. Raglio *et al.* (Raglio *et al.*, 2016) reported a statistically significant decrease in anxiety across the intervention. Two studies reported that State Anxiety was statistically significantly lower post-intervention (Brotons and Marti, 2003; García-Valverde *et al.*, 2020). No significant differences were obtained in the Trait Anxiety dimension in the García-Valverde *et al.* (García-Valverde *et al.*, 2020) study and Trait Anxiety was not reported in Brotons and Marti (Brotons and Marti, 2003). Baker *et al.* (Baker *et al.*, 2012) reported no significant change in anxiety. Lack of significance was attributed to the small sample size and the low levels of anxiety recorded pre-and post-intervention (Baker *et al.*, 2012). Change in depression was measured in nine studies; using four different measures (Supplementary materials D and E). Statistical significance was only found in one study (García-Valverde *et al.*, 2020).

Eleven studies measured changes in well-being or quality of life, or specific aspect(s) of these constructs (Supplementary materials D and E). Both García-Valverde *et al.* (García-Valverde *et al.*, 2020) and Mittelman and Papayannopoulou (Mittelman and Papayannopoulou, 2018) reported a statistically significant improvement in self-esteem across the intervention. There were also statistically significant increases in positive mood in a study by Davidson and Almeida (Davidson and Almeida, 2014) and in relaxation, comfort and happiness in a study by Hanser *et al.* (Hanser *et al.*, 2011). García-Valverde *et al.* (García-Valverde *et al.*, 2020) also reported that the mean post-test scores were statistically significantly higher in the Mental Component Summary and the Mental Health dimension of the Spanish version of the Short-Form Health Survey (SF-36v2) utilized. No other statistically significant findings were reported.

Twelve studies employed measures directly related to caring (Supplementary materials D and E). Clair and Ebberts (Clair and Ebberts, 1997) reported a statistically significant increase in satisfaction with visits across the intervention. Raglio *et al.* (Raglio *et al.*, 2016) and Särkämö *et al.* (Särkämö *et al.*, 2014) both reported statistically significant decreases in carer burden post-intervention. Särkämö *et al.* (Särkämö *et al.*, 2014) observed a greater decrease in burden in the singing group (*p* = 0.026) than the music listening (*p* = 0.029) or control (*p* = 0.069) groups. No other statistically significant findings were reported.

## DISCUSSION

The results of the integrative review provide evidence that music interventions may improve social and emotional well-being, ameliorate the caring relationship and benefit coping and caring for family carers.

Family carers commonly experience feelings of social isolation and loneliness (Tamplin *et al.*, 2018). A report published by Family Carers Ireland (Family Carers Ireland, 2020) suggests that a significant number of family carers in Ireland receive limited support from the State, other family member and friends, and primarily care alone. Further, an analysis of carer burden among family carers of people with and without dementia in Ireland found dementia diagnosis to be significantly associated with carer burden (Teahan *et al.*, 2021). The results of this integrative review suggest that music interventions can provide family carers with the opportunity to meet other carers who can relate to what they are experiencing, and receive empathetic support. They can stimulate feelings of belonging and solidarity, and alleviate burden.

The role that peer support can play in enabling family carers to cope better with challenges and continue caring is highlighted in the literature (Smith *et al.*, 2018). Social connection is a common goal, and reported benefit, of support groups and psychosocial interventions for family carers (Teahan *et al.*, 2020a). However, this review identifies that music interventions can offer social experiences that are distinct from other interventions, where focus tends to be on information sharing and addressing day-to-day challenges (Teahan *et al.*, 2020a). Music is inherently relational. It is a powerful trigger of nostalgia, and enables people to access shared culture, experiences, aesthetics and emotions (Barrett *et al.*, 2010). In this review, singing emerged as the primary musical medium utilized across the interventions, from music therapy, to choirs, to songwriting groups. Therapeutic songwriting interventions were found to facilitate emotional expression. They enabled family carers to express their thoughts and feelings around being a carer in a supportive environment, and allowed them to focus on the positive aspects of caring. Group singing interventions for caring dyads offered a different environment for social support by providing an opportunity for family carers to engage in an accessible social activity with their care recipient. A mixed methods systematic review by Bressan *et al.* (Bressan *et al.*, 2020) highlights that, in addition to education opportunities and dementia care skills training, family carers need social, psychological and emotional support. This review suggests that music interventions may be an effective means of providing this.

The progression of dementia can severely compromise the quality of the relationship between the person living with dementia and their family carer, augmenting feelings of isolation and loneliness. The results of this integrative review demonstrate how music can ameliorate the caring relationship. A study by Morrisby *et al.* (Morrisby *et al.*, 2019) discussed the importance of developing and maintaining strong caring relationships in dementia. It identified ‘love, humour, patience and tolerance’ as key attitudes [(Morrisby *et al.*, 2019), p. e47]. Haire and MacDonald [(Haire and MacDonald, 2021), p. 3] similarly identified humour as a ‘connective lifeline’. However, as dementia progresses, one of the biggest challenges reported by family carers is the increasing lack of awareness that the care recipient may have into how much their carer is supporting them. The ability of music interventions to ‘bring back’ the person living with dementia and strengthen reciprocity within the dyad can be seen to be valuable in maintaining a positive caring relationship throughout the dementia journey and promoting carer well-being. Having a positive relationship with the person living with dementia is recognized as a positive mediator in Daley *et al.*’s (Daley *et al.*, 2019) conceptual framework for understanding the quality of life of family carers of people living with dementia.

A notable benefit of many of the music interventions included in this review was the potential for family carers to continue using music beyond the intervention. This is particularly important for sustaining the effects and support of carer independence and resilience. Building carer resilience is seen as central to the maintenance and sustenance of family care of people living with dementia (Parkinson *et al.*, 2017). The findings of this review also suggest that music interventions may contribute to experiences of flourishing for family carers (Seligman, 2011). Seligman’s criteria for flourishing include ‘being in the upper range of positive emotion, engagement, positive relationships, meaning, and positive accomplishment’ [(Hone *et al.*, 2014), p. 70]. The findings suggest that family carers may have the opportunity to fulfil these criteria and experience flourishing through engaging in a music intervention.

The findings of this review suggest that family carers’ perceptions of music interventions are generally positive. They described them as enjoyable and beneficial, highlighting aspects that helped to make them accessible for their care recipient and themselves. The subject of cost of participation was not raised, despite the widely reported financial struggles of family carers (Family Carers Ireland, 2020). Notably, many of the features of enjoyment mentioned are completely independent of the musical engagement itself. The finding that some participants were initially apprehensive about engaging, identifying as ‘non-musical’, reflects research in other areas on singing attitudes and confidence in adults and is important to note as it may have implications for the process of recruitment in future studies.

Rich qualitative findings provide insight into the impact of music interventions on family carers, and their perceptions of them. However, of the 50 psychological measures (34 different psychological tools) used across the studies to investigate the impact of participation on an aspect, or multiple aspects, of the family carers’ health and well-being, statistical significance was only achieved in 13 cases. Overall, we found that music interventions were deeply meaningful to carers, but often statistically insignificant.

Null quantitative results, however, should not be equated with the absence of results (Franco *et al.*, 2014; Miller-Halegoua, 2017; Visentin *et al.*, 2020). The prevalence of null findings may be attributable to the small sample sizes utilized, typical of the feasibility studies included. Furthermore, the impact of intervention duration on quantitative findings should be considered (see Supplementary material F) and the potential impact of longer music interventions explored. Researchers should draw on the findings of the high-quality, small-scale, studies included in this review when designing larger studies in this area. Reported effect sizes and associated qualitative data may shed light on the sensitivity and suitability of psychological tools. For example, only one validated instrument designed specifically for dementia carers was utilized. Similarly, challenges reported in relation to conducting research in this area, such as issues with recruitment, or the intervention itself, can be addressed. It is important to note that the statistically significant findings indicated that music interventions positively impacted aspects of health and well-being. They were consistent with the qualitative findings, and no adverse quantitative findings were reported. In addition, it is worth considering whether maintenance, rather than improvement, over time is a more realistic goal when dealing with a degenerative condition.

This integrative review has limitations. Only studies written in English were included and, as a result, studies from other geographical regions and linguistic groups may have been excluded. Only 342 carers were included in this integrative review, which limits generalizability. The findings of the integrative review highlight the need for larger, rigorous studies in this area, such as further research into music interventions for family carers to attend without their care recipients. The descriptive results also lend an insight into the lack of participant characteristics commonly reported. Future research should include these participant characteristics to facilitate data synthesis and sub-group analyses. Lastly, the potential of biased samples should be recognized as participants in these studies were not blinded to the type of intervention and may have consented to take part due to an existing interest in music or preconceptions about its value.

In conclusion, this integrative review provides support for the provision of music interventions for family carers of people living with dementia. It indicates that they may improve family carers’ social and emotional well-being, enhance their ability to cope and care and ameliorate the caring relationship, contributing to experiences of flourishing. It contributes to the growing research evidence of the benefits of singing for health and well-being. However, it highlights that this area is under-researched and points to the need for larger, more rigorous studies.

## SUPPLEMENTARY MATERIAL

Supplementary material is available at *Health Promotion International* online.

## FUNDING

This work was supported by the Irish Research Council [grant number GOIPG/2019/3222] and the National Institute on Aging at the U.S. National Institutes of Health [grant number K23AG062613].

## CONFLICT OF INTEREST STATEMENT

The authors report no conflict of interest.

## Supplementary Material

daac024_Supplementary_Data
