# Supplementary material for: Integrative review of singing and music interventions for family carers of people living with dementia
Source: Health Promot Int. 2022 Apr 13;37(Suppl 1):i49–61. doi: 10.1093/heapro/daac024 (PMC9162174; doi:10.1093/heapro/daac024)
Supplement: daac024_Supplementary_Data [file daac024_supplementary_data.zip › daac024-suppl_data/Supplementary Material A.docx]

**Supplementary Material A: Database Search Strategies**

**Table S1a**

*Search Strategy for Medline/Ovid*

| 1 | exp Dementia/ | 169002 |
| --- | --- | --- |
| 2 | Delirium/ | 9540 |
| 3 | Wernicke Encephalopathy/ | 1748 |
| 4 | Delirium, Dementia, Amnestic, Cognitive Disorders/ | 9281 |
| 5 | dement*.mp. | 135060 |
| 6 | alzheimer*.mp. | 164831 |
| 7 | (lewy* adj2 bod*).mp. | 10402 |
| 8 | deliri*.mp. | 19416 |
| 9 | (chronic adj2 cerebrovascular).mp. | 666 |
| 10 | ("organic brain disease" or "organic brain syndrome").mp. | 796 |
| 11 | ("normal pressure hydrocephalus" and "shunt*").mp. | 1474 |
| 12 | "benign senescent forgetfulness".mp. | 18 |
| 13 | (cerebr* adj2 deteriorat*).mp. | 238 |
| 14 | (cerebral* adj2 insufficient*).mp. | 84 |
| 15 | (pick* adj2 disease).mp. | 3875 |
| 16 | (creutzfeldt or jcd or cjd).mp. | 8755 |
| 17 | huntington*.mp. | 19868 |
| 18 | binswanger*.mp. | 592 |
| 19 | korsako*.mp. | 1710 |
| 20 | 1 or 2 or 3 or 4 or 5 or 6 or 7 or 8 or 9 or 10 or 11 or 12 or 13 or 14 or 15 or 16 or 17 or 18 or 19 | 306952 |
| 21 | family.ab. | 748263 |
| 22 | "spous*".ab. | 18715 |
| 23 | partner.ab. | 71252 |
| 24 | "home*".ab. | 457864 |
| 25 | "informal care*" | 5238 |
| 26 | 21 or 22 or 23 or 24 or 25 | 1235343 |
| 27 | "music*".ab. | 18252 |
| 28 | exp Music Therapy/ | 3604 |
| 29 | singing.ab. | 2848 |
| 30 | sing.ab. | 1086 |
| 31 | "auditory stimul* ".ab. | 7074 |
| 32 | piano.ab. | 1234 |
| 33 | "song*".ab. | 9992 |
| 34 | choir.ab. | 229 |
| 35 | chorus.ab. | 436 |
| 36 | choral.ab. | 149 |
| 37 | 27 or 28 or 29 or 30 or 31 or 32 or 33 or 34 or 35 or 36 | 38061 |
| 38 | 20 and 26 | 31218 |
| 39 | 37 and 38 | 242 |
| 40 | limit 39 to english language | 232 |

**Table S1b**

*Search Strategy for Psycinfo/ EBSCO*

| S1 | MA DE "Dementia" OR DE "AIDS Dementia Complex" OR DE "Dementia with Lewy Bodies" OR DE "Presenile Dementia" OR DE "Pseudodementia" OR DE "Semantic Dementia" OR DE "Senile Dementia" OR DE "Vascular Dementia" | 46,691 |
| --- | --- | --- |
| S2 | MA Delirium | 2,736 |
| S3 | MA Wernickes Encephalopathy | 217 |
| S4 | TX dement* | 82,034 |
| S5 | TX alzheimer* | 71,280 |
| S6 | TX lewy* N2 bod* | 4,168 |
| S7 | TX deliri* | 8,225 |
| S8 | TX chronic N2 cerebrovascular | 122 |
| S9 | TX "organic brain disease" OR "organic brain syndrome" | 866 |
| S10 | TX "normal pressure hydrocephalus" AND "shunt*" | 238 |
| S11 | TX "benign senescent forgetfulness" | 27 |
| S12 | TX cerebr* N2 deteriorat* | 72 |
| S13 | TX cerebral* N2 insufficient* | 10 |
| S14 | TX pick* N2 disease | 825 |
| S15 | TX creutzfeldt OR jcd OR cjd | 1,251 |
| S16 | TX huntington* | 10,141 |
| S17 | TX binswanger* | 615 |
| S18 | TX korsako* | 2,975 |
| S19 | S1 OR S2 OR S3 OR S4 OR S5 OR S6 OR S7 OR S8 OR S9 OR S10 OR S11 OR S12 OR S13 OR S14 OR S15 OR S16 OR S17 OR S18 | 134,740 |
| S20 | AB family | 361,419 |
| S21 | AB spous* | 20,785 |
| S22 | AB partner | 83,033 |
| S23 | AB home* | 154,515 |
| S24 | AB "informal care*" | 3,228 |
| S25 | S20 OR 21 OR S22 OR S23 OR S24 | 555,281 |
| S26 | AB music* | 35,590 |
| S27 | MA Music Therapy | 1,012 |
| S28 | AB singing | 3,651 |
| S29 | AB sing | 1,264 |
| S30 | AB "auditory stimul* " | 6,646 |
| S31 | AB piano | 1,510 |
| S32 | AB song* | 8,784 |
| S33 | AB choir | 389 |
| S34 | AB chorus | 654 |
| S35 | AB choral | 405 |
| S36 | S26 OR S27 OR S28 OR S29 OR S30 OR S31 OR S32 OR S32 OR S33 OR S34 OR S35 | 51,066 |
| S37 | S19 AND S25 | 20,903 |
| S38 | S36 AND S37 | 259 |
| S39 | Narrowed by Language - English | 243 |

**Table S1c**

*Search Strategy for CINAHL/ EBSCO*

| S1 | MH "Dementia+" | 74,659 |
| --- | --- | --- |
| S2 | MH "Delirium" | 6,753 |
| S3 | MH "Wernicke’s Encephalopathy" | 441 |
| S4 | TX dement* | 119,767 |
| S5 | TX alzheimer* | 83,643 |
| S6 | TX lewy* N2 bod* | 4,488 |
| S7 | TX deliri* | 21,599 |
| S8 | TX chronic N2 cerebrovascular | 732 |
| S9 | TX "organic brain disease" OR "organic brain syndrome" | 368 |
| S10 | TX "normal pressure hydrocephalus" AND "shunt*" | 230 |
| S11 | TX "benign senescent forgetfulness" | 37 |
| S12 | TX cerebr* N2 deteriorat* | 184 |
| S13 | TX cerebral* N2 insufficient* | 53 |
| S14 | TX pick* N2 disease | 1,211 |
| S15 | TX creutzfeldt OR jcd OR cjd | 2,874 |
| S16 | TX huntington* | 30,365 |
| S17 | TX binswanger* | 651 |
| S18 | TX korsako* | 948 |
| S19 | S1 OR S2 OR S3 OR S4 OR S5 OR S6 OR S7 OR S8 OR S9 OR 10 OR 11 OR 12 OR 13 OR 14 OR 15 OR 16 OR 17 OR 18 | 208,064 |
| S20 | AB family | 211,155 |
| S21 | AB spous* | 9,535 |
| S22 | AB partner | 46,052 |
| S23 | AB home* | 145,574 |
| S24 | AB "informal care*" | 3,965 |
| S25 | S20 OR S21 OR S22 OR S23 OR S24 | 374,898 |
| S26 | AB music* | 9,890 |
| S27 | MH “Music Therapy” | 5,968 |
| S28 | AB singing | 1,335 |
| S29 | AB sing | 375 |
| S30 | AB "auditory stimul* " | 913 |
| S31 | AB piano | 296 |
| S32 | AB song* | 1,617 |
| S33 | AB choir | 161 |
| S34 | AB chorus | 97 |
| S35 | AB choral | 96 |
| S36 | S26 OR S27 OR S28 OR S29 OR S30 OR S31 OR 32 OR 32 OR 33 OR 34 OR 35 | 15,682 |
| S37 | S19 AND S25 | 22,344 |
| S38 | S36 AND S37 | 318 |
| S39 | Narrowed by Language – English | 311 |

**Table S1d**

*Search Strategy for Academic Search Complete*

| S1 | TX dement* | 233,842 |
| --- | --- | --- |
| S2 | TX alzheimer* | 233,001 |
| S3 | TX Wernicke’s Encephalopathy | 1,591 |
| S4 | TX lewy* N2 bod* | 15,379 |
| S5 | TX deliri* | 47,398 |
| S6 | TX chronic N2 cerebrovascular | 2,176 |
| S7 | TX "organic brain disease" OR "organic brain syndrome" | 9,450 |
| S8 | TX "normal pressure hydrocephalus" AND "shunt*" | 625 |
| S9 | TX "benign senescent forgetfulness" | 75 |
| S10 | TX cerebr* N2 deteriorat* | 529 |
| S11 | TX cerebral* N2 insufficient* | 183 |
| S12 | TX pick* N2 disease | 6,304 |
| S13 | TX creutzfeldt OR jcd OR cjd | 11,223 |
| S14 | TX huntington* | 87,950 |
| S15 | TX binswanger* | 3,424 |
| S16 | TX korsako* | 10,143 |
| S17 | S1 OR S2 OR S3 OR S4 OR S5 OR S6 OR S7 OR S8 OR S9 OR 10 OR 11 OR 12 OR 13 OR 14 OR 15 OR 16 | 495,065 |
| S18 | AB family | 880,352 |
| S19 | AB spous* | 21,313 |
| S20 | AB partner | 199,397 |
| S21 | AB home* | 586,211 |
| S22 | AB "informal care*" | 3,894 |
| S23 | S18 OR S19 OR S20 OR 21 OR S22 | 1,595,096 |
| S24 | AB music* | 562,511 |
| S25 | AB “Music Therapy” | 2,229 |
| S26 | AB singing | 15,168 |
| S27 | AB sing | 10,687 |
| S28 | AB "auditory stimul* " | 4,010 |
| S29 | AB piano | 28,693 |
| S30 | AB song* | 120,596 |
| S31 | AB choir | 9,721 |
| S32 | AB chorus | 6,054 |
| S33 | AB choral | 7,929 |
| S34 | S24 OR S25 OR S26 OR S27 OR S28 OR S29 OR S30 OR S31 OR S32 OR S33 | 665,801 |
| S35 | S17 AND S23 | 45,667 |
| S36 | S34 AND S35 | 501 |
| S37 | Narrowed by Language – English | 481 |

**Table S1e**

*Search Strategy for SCOPUS*

| 1 | ALL (dement*) | 621,799 |
| --- | --- | --- |
| 2 | ALL (alzheimer*) | 733,966 |
| 3 | ALL ("Wernicke Encephalopathy") | 6,532 |
| 4 | ALL (lewy* W/2 bod*) | 57,679 |
| 5 | ALL (deliri*) | 73,641 |
| 6 | ALL (chronic W/2 cerebrovascular) | 3,657 |
| 7 | (ALL ("organic brain disease") OR ALL ("organic brain syndrome")) | 7,359 |
| 8 | (ALL ("normal pressure hydrocephalus") AND ALL ("shunt*")) | 4,762 |
| 9 | ALL ("benign senescent forgetfulness") | 226 |
| 10 | ALL (cerebr* W/2 deteriorat*) | 1,417 |
| 11 | ALL (cerebr* W/2 insufficient*) | 195 |
| 12 | ALL (pick* W/2 disease) | 22,904 |
| 13 | (ALL (creutzfeldt) OR ALL (jcd) OR ALL (cjd)) | 62,600 |
| 14 | ALL (huntington*) | 263,259 |
| 15 | ALL (binswanger*) | 22,407 |
| 16 | ALL (korsako*) | 50,430 |
| 17 | #1 OR #2 OR #3 OR #4 OR #5 OR #6 OR #7 OR #8 OR #9 OR #10 OR #11 OR #12 OR #13 OR #14 OR #15 OR #16 | 1,408,244 |
| 18 | ABS (family) | 1,565,186 |
| 19 | ABS (spous*) | 31,032 |
| 20 | ABS (partner) | 304,258 |
| 21 | ABS (home*) | 795,824 |
| 22 | ABS ("informal care*") | 6,790 |
| 23 | #18 OR #19 OR #20 OR #21 OR #22 | 2,557,705 |
| 24 | ABS (music*) | 121,636 |
| 25 | ABS ("music therapy") | 4,162 |
| 26 | ABS (singing) | 10,194 |
| 27 | ABS (sing) | 6,760 |
| 28 | ABS ("auditory stimul*") | 9,641 |
| 29 | ABS (piano) | 6,766 |
| 30 | ABS (song*) | 50,952 |
| 31 | ABS (choir) | 1,652 |
| 32 | ABS (chorus) | 3,835 |
| 33 | ABS (choral) | 1,195 |
| 34 | #24 OR #25 OR #26 OR #27 OR #28 OR #29 OR #30 OR #31 OR #32 OR #33 | 187,553 |
| 35 | #17 AND #23 | 133,578 |
| 36 | #34 AND 35 | 700 |
| 37 | #42 English Only | 659 |

**Table S1f**

*Search Strategy for Web of Science*

| 1 | ALL=dement* | 190,828 |
| --- | --- | --- |
| 2 | ALL=alzheimer* | 270,376 |
| 3 | ALL=("Wernicke Encephalopathy") | 543 |
| 4 | TS=(lewy* NEAR/2 bod*) | 15,155 |
| 5 | ALL=deliri* | 19,965 |
| 6 | TS=(chronic NEAR/2 cerebrovascular) | 769 |
| 7 | ALL=("organic brain disease") OR ALL=("organic brain syndrome") | 561 |
| 8 | ALL=("normal pressure hydrocephalus") AND ALL=("shunt*") | 1,547 |
| 9 | ALL=("benign senescent forgetfulness") | 27 |
| 10 | TS=(cerebr* NEAR/2 deteriorat*) | 308 |
| 11 | TS=(cerebr* NEAR/2 insufficient*) | 93 |
| 12 | TS=(pick* NEAR/2 disease) | 5,092 |
| 13 | ALL=(creutzfeldt) OR ALL=(jcd) OR ALL=(cjd) | 18,766 |
| 14 | ALL=huntington* | 73,239 |
| 15 | ALL=binswanger* | 1,634 |
| 16 | ALL=korsako* | 17,151 |
| 17 | #1 OR #2 OR #3 OR #4 OR #5 OR #6 OR #7 OR #8 OR #9 OR #10 OR #11 OR #12 OR #13 OR #14 OR #15 OR #16 | 489,125 |
| 18 | AB=family | 1,133,269 |
| 19 | AB=spous* | 20,686 |
| 20 | AB=partner | 202,782 |
| 21 | AB=home* | 557,922 |
| 22 | AB=("informal care*") | 5,329 |
| 23 | #18 OR #19 OR #20 OR #21 OR #22 | 1,818,446 |
| 24 | AB=music* | 75,637 |
| 25 | AB=("music therapy") | 2,397 |
| 26 | AB=singing | 13,987 |
| 27 | AB=sing | 13,124 |
| 28 | AB=("auditory stimul*") | 6,666 |
| 29 | AB=piano | 4,804 |
| 30 | AB=song* | 31,392 |
| 31 | AB=choir | 1,171 |
| 32 | AB=chorus | 2,965 |
| 33 | AB=choral | 832 |
| 34 | #24 OR #25 OR #26 OR #27 OR #28 OR #29 OR #30 OR #31 OR #32 OR #33 | 120,394 |
| 35 | #17 AND #23 | 44,323 |
| 36 | #34 AND #35 | 316 |
| 37 | (#34 AND #35) AND LANGUAGE: (English) | 306 |

*Search Strategy for Sage*

[[All dementi*] OR [All alzheimer*] OR [All "wernicke encephalopathy"] OR [All "lewy* bod*"] OR [All deliri*] OR [All "chronic cerebrovascular"] OR [All "organic brain disease"] OR [All "organic brain syndrome"] OR [All "benign senescent forgetfulness"] OR [All "cerebr* deteriorat*"] OR [All "cerebral* insufficient*"] OR [All "pick* disease"] OR [All creutzfeldt] OR [All jcd] OR [All cjd] OR [All huntington*] OR [All binswanger*] OR [All korsako*] OR [[All "normal pressure hydrocephalus"] AND [All "shunt*"]]] AND [[Abstract family] OR [Abstract spous*] OR [Abstract partner] OR [Abstract home*] OR [Abstract "informal care*"]] AND [[Abstract music*] OR [Abstract "music therapy"] OR [Abstract singing] OR [Abstract song] OR [Abstract "auditory stimul*"] OR [Abstract piano] OR [Abstract song*] OR [Abstract choir] OR [Abstract chorus] OR [Abstract choral]] 208

*Search Strategies for Science Direct*

#1 Title, abstract, keywords: (dementia OR alzheimer) AND (family OR spouse OR partner OR care) AND (music OR singing OR song) 84

#2 Title, abstract, keywords: (dementia OR alzheimer) AND (family OR spouse OR partner OR home) AND (music OR singing OR song) 55

**Table S1g**

*Search Strategy for AMED/EBSCO*

| S1 | TX dement* | 2,906 |
| --- | --- | --- |
| S2 | TX alzheimer* | 1,491 |
| S3 | TX Wernicke’s Encephalopathy | 4 |
| S4 | TX lewy* N2 bod* | 25 |
| S5 | TX deliri* | 277 |
| S6 | TX chronic N2 cerebrovascular | 3 |
| S7 | TX "organic brain disease" OR "organic brain syndrome" | 4 |
| S8 | TX "normal pressure hydrocephalus" AND "shunt*" | 15 |
| S9 | TX "benign senescent forgetfulness" | 0 |
| S10 | TX cerebr* N2 deteriorat* | 1 |
| S11 | TX cerebral* N2 insufficient* | 0 |
| S12 | TX pick* N2 disease | 3 |
| S13 | TX creutzfeldt OR jcd OR cjd | 16 |
| S14 | TX huntington* | 271 |
| S15 | TX binswanger* | 5 |
| S16 | TX korsako* | 18 |
| S17 | S1 OR S2 OR S3 OR S4 OR S5 OR S6 OR S7 OR S8 OR S9 OR 10 OR 11 OR 12 OR 13 OR 14 OR 15 OR 16 | 4,410 |
| S18 | AB family | 9,836 |
| S19 | AB spous* | 574 |
| S20 | AB partner | 941 |
| S21 | AB home* | 10,564 |
| S22 | AB "informal care*" | 294 |
| S23 | S18 OR S19 OR S20 OR S21 OR S22 | 19,898 |
| S24 | AB music* | 875 |
| S25 | AB “Music Therapy” | 340 |
| S26 | AB singing | 56 |
| S27 | AB sing | 31 |
| S28 | AB "auditory stimul* " | 81 |
| S29 | AB piano | 15 |
| S30 | AB song* | 100 |
| S31 | AB choir | 9 |
| S32 | AB chorus | 4 |
| S33 | AB choral | 13 |
| S34 | S24 OR S25 OR S26 OR S27 OR S28 OR S29 OR S30 OR S31 OR S32 OR S33 | 1,083 |
| S35 | S17 AND S23 | 739 |
| S36 | S34 AND S35 | 24 |
| S37 | Narrowed by Language – English | 24 |

**Table S1h**

*Search Strategy for Cochrane*

| #1 | MeSH descriptor: [Dementia] explode all trees | 5935 |
| --- | --- | --- |
| #2 | MeSH descriptor: [Delirium] explode all trees | 769 |
| #3 | MeSH descriptor: [Wernicke Encephalopathy] explode all trees | 4 |
| #4 | (dement*) | 23779 |
| #5 | (alzheimer*) | 12440 |
| #6 | (lewy* NEAR/2 bod*) | 537 |
| #7 | (deliri*) | 3862 |
| #8 | (chronic NEAR/2 cerebrovascular) | 603 |
| #9 | ("organic brain disease") OR ("organic brain syndrome") | 242 |
| #10 | ("normal pressure hydrocephalus") AND ("shunt*") | 130 |
| #11 | ("benign senescent forgetfulness") | 78 |
| #12 | (cerebr* NEAR/2 deteriorat*) | 96 |
| #13 | (cerebral* NEAR/2 insufficient*) | 68 |
| #14 | (pick* NEAR/2 disease) | 146 |
| #15 | (creutzfeldt) OR (jcd) OR (cjd) | 286 |
| #16 | (huntington*) | 1152 |
| #17 | (binswanger*) | 106 |
| #18 | (korsako*) | 765 |
| #19 | #1 OR #2 OR #3 OR #4 OR #5 OR #6 OR #7 OR #8 OR #9 OR #10 OR #11 OR #12 OR #13 OR #14 OR #15 OR #16 OR #17 OR #18 | 32055 |
| #20 | (family):ab | 25881 |
| #21 | (spous*):ab | 1512 |
| #22 | (partner):ab | 6091 |
| #23 | (home*):ab | 50214 |
| #24 | ("informal care*"):ab | 190 |
| #25 | #20 OR #21 OR #22 OR #23 OR #24 | 76947 |
| #26 | (Music*):ab | 3940 |
| #27 | MeSH descriptor: [Music Therapy] explode all trees | 824 |
| #28 | (singing):ab | 311 |
| #29 | (sing):ab | 100 |
| #30 | (auditory NEXT stimul*):ab | 608 |
| #31 | (piano):ab | 78 |
| #32 | (song*):ab | 433 |
| #33 | (choir):ab | 37 |
| #34 | (chorus):ab | 33 |
| #35 | (choral):ab | 30 |
| #36 | #26 OR #27 OR #28 OR #29 OR #30 OR #31 OR #32 OR #33 OR #35 OR #36 OR #37 OR #38 | 5055 |
| #37 | #19 AND #25 | 4301 |
| #38 | #37 AND #38 | 132 |

**Table S1i**

*Search Strategy for PubMed*

| 1 | dementia[MeSH Terms] | 169,086 |
| --- | --- | --- |
| 2 | delirium[MeSH Terms] | 9,762 |
| 3 | Wernicke Encephalopathy[MeSH Terms] | 1,748 |
| 4 | dement* | 144,980 |
| 5 | alzheimer* | 174,982 |
| 6 | (lewy*) AND (body) | 6,372 |
| 7 | (lewy*) AND (bodies) | 8,521 |
| 8 | deliri* | 19,451 |
| 9 | (chronic) AND (cerebrovascular) | 11,533 |
| 10 | ("organic brain disease") OR ("organic brain syndrome") | 797 |
| 11 | ("normal pressure hydrocephalus") AND ("shunt*") | 1,668 |
| 12 | "benign senescent forgetfulness" | 18 |
| 13 | (cerebr*) AND (deteriorat*) | 10,570 |
| 14 | (cerebral*) AND (insufficient*) | 2,527 |
| 15 | (pick*) AND (disease) | 14,445 |
| 16 | ((creutzfeldt) OR (jcd)) OR (cjd) | 12,062 |
| 17 | huntington* | 30,676 |
| 18 | binswanger* | 1,068 |
| 19 | korsako* | 21,421 |
| 20 | #1 OR #2 OR #3 OR #4 OR #5 OR #6 OR #7 OR #8 OR #9 OR #10 OR #11 OR #12 OR #13 OR #14 OR #15 OR #16 OR #17 OR #18 OR #19 | 375,745 |
| 21 | Family[Title/Abstract] | 830,771 |
| 22 | spous*[Title/Abstract] | 19,821 |
| 23 | partner[Title/Abstract] | 77,536 |
| 24 | home*[Title/Abstract] | 511,990 |
| 25 | "informal care*"[Title/Abstract] | 5,804 |
| 26 | #21 OR #22 OR #23 OR #24 OR #25 | 1,372,915 |
| 27 | Music*[Title/Abstract] | 22,029 |
| 28 | "music therapy"[MeSH Terms] | 3,606 |
| 29 | singing[Title/Abstract] | 3,326 |
| 30 | sing[Title/Abstract] | 1,322 |
| 31 | "auditory stimul* "[Title/Abstract] | 7,762 |
| 32 | piano[Title/Abstract] | 1,307 |
| 33 | song*[Title/Abstract] | 11,544 |
| 34 | choir[Title/Abstract] | 383 |
| 35 | chorus[Title/Abstract] | 519 |
| 36 | choral[Title/Abstract] | 172 |
| 37 | #27 OR #28 OR #29 OR #30 OR #31 OR #32 OR #33 OR #34 OR #35 OR #36 | 43,923 |
| 38 | #20 AND #26 | 37,129 |
| 39 | #37 AND #38 | 269 |
| 40 | #39 English Only | 257 |
