# Supplementary material for: Integrative review of singing and music interventions for family carers of people living with dementia
Source: Health Promot Int. 2022 Apr 13;37(Suppl 1):i49–61. doi: 10.1093/heapro/daac024 (PMC9162174; doi:10.1093/heapro/daac024)
Supplement: daac024_Supplementary_Data [file daac024_supplementary_data.zip › daac024-suppl_data/Supplementary Material C.docx]

**Supplementary Material C: Mixed Methods Appraisal Tool Results**

**Table S2a**

*Mixed Methods Appraisal Tool: Qualitative*

|  | Q1 | Q2 | Q3 | Q4 | Q5 |
| --- | --- | --- | --- | --- | --- |
| Baker & Yeates, 2018 | Y | Y | Y | Y | Y |
| Dassa et al., 2020 | Y | Y | ? | Y | Y |
| Dowlen, 2018 | Y | Y | Y | Y | Y |
| Dupuis & Pedlar, 1995 | Y | Y | Y | Y | Y |
| Garabedian & Kelly, 2020 | Y | Y | ? | Y | Y |
| Gardner, 1999 | Y | Y | Y | Y | Y |
| Hanser & Clair, 1995 | Y | ? | ? | ? | ? |
| Harris & Caporella, 2014 | Y | Y | Y | Y | Y |
| Harris & Caporella, 2019 | Y | Y | Y | Y | Y |
| Lee et al., 2020 | Y | Y | Y | Y | Y |
| Macgregor, 2016 | Y | Y | Y | Y | Y |
| Osman et al., 2016 | Y | Y | Y | Y | Y |
| Shibazaki & Marshall, 2017 | Y | Y | N | Y | Y |
| Unadkat et al., 2017 | Y | Y | Y | Y | Y |

Q1. Is the qualitative approach appropriate to answer the research question?

Q2. Are the qualitative data collection methods adequate to address the research question?

Q3. Are the findings adequately derived from the data?

Q4. Is the interpretation of results sufficiently substantiated by data?

Q5. Is there coherence between qualitative data sources, collection, analysis and interpretation?

**Table S2b**

*Mixed Methods Appraisal Tool: Quantitative Randomised Controlled Trials*

|  | Q1 | Q2 | Q3 | Q4 | Q5 |
| --- | --- | --- | --- | --- | --- |
| Särkämö et al., 2014 | Y | N* | Y | Y | Y** |

Q1. Is randomization appropriately performed?

Q2. Are the groups comparable at baseline?

Q3. Are there complete outcome data?

Q4. Are outcome assessors blinded to the intervention provided?

Q5 Did the participants adhere to the assigned intervention?

*Group differences were controlled for statistically in the longitudinal group comparisons (Särkämö et al., 2014).

** Engagement in group sessions and homework was high during the 10-week intervention. At Follow-up 2, 68% of the carers reported having used the trained musical activities with their care recipients at least once a week.

**Table S2c**

*Mixed Methods Appraisal Tool: Quantitative Non-Randomised*

|  | Q1 | Q2 | Q3 | Q4 | Q5 |
| --- | --- | --- | --- | --- | --- |
| Brotons & Marti, 2003 | N | Y | Y | N | Y |
| Clair & Ebberts, 1997 | N | Y | Y | N | Y |
| García-Valverde et al., 2020 | N | Y | Y | N | Y |
| Holden et al., 2019 | N | Y | N | N | Y |
| Klein & Silverman, 2012 | N | Y | ? | N | Y |
| Raglio et al., 2016 | N | Y | Y | N | Y |

Q1. Are the participants representative of the target population?

Q2. Are measurements appropriate regarding both the outcome and intervention (or exposure)?

Q3. Are there complete outcome data?

Q4. Are the confounders accounted for in the design and analysis?

Q5. During the study period, is the intervention administered (or exposure occurred) as intended?

**Table S2d**

*Mixed Methods Appraisal Tool: Mixed Methods*

|  | Q1 | Q2 | Q3 | Q4 | Q5 |
| --- | --- | --- | --- | --- | --- |
| Baker et al., 2012 | Y | Y | Y | Y | N |
| Baker et al., 2018 | Y | Y | Y | Y | Y |
| Camic et al., 2013 | N | N | N | Y | N |
| Clair, 2002 | N | N | N | N | N |
| Clair et al., 1993 | N | Y | Y | Y | N |
| Clark et al., 2020 | N | Y | Y | Y | Y |
| Davidson & Almeida, 2014 | Y | Y | Y | Y | N |
| Hanser et al., 2011 | N | Y | Y | Y | N |
| Melhuish et al., 2019 | N | Y | Y | Y | N |
| Mittelman & Papayannopoulou, 2018 | Y | Y | Y | Y | N |
| Särkämö et al., 2013; Särkämö et al., 2014 | N | Y | Y | Y | Y |
| Tamplin et al., 2018; Clark et al., 2018 | Y | ?* | ?* | ?* | Y |
| Zeilig et al., 2019 | Y | Y | Y | Y | Y |

Q1. Is there an adequate rationale for using a mixed methods design to address the research question?

Q2. Are the different components of the study effectively integrated to answer the research question?

Q3. Are the outputs of the integration of qualitative and quantitative components adequately interpreted?

Q4. Are divergences and inconsistencies between quantitative and qualitative results adequately addressed?

Q5. Do the different components of the study adhere to the quality criteria of each tradition of the methods involved?

* Quantitative and qualitative findings are reported in different publications
