# Supplementary material for: Integrative review of singing and music interventions for family carers of people living with dementia
Source: Health Promot Int. 2022 Apr 13;37(Suppl 1):i49–61. doi: 10.1093/heapro/daac024 (PMC9162174; doi:10.1093/heapro/daac024)
Supplement: daac024_Supplementary_Data [file daac024_supplementary_data.zip › daac024-suppl_data/Supplementary Material D.docx]

**Supplementary Material D: Quantitative Psychological Measurement Tools for Health and Well-being**

**Table S3**

*Quantitative Psychological Measurement Tools for Health and Well-being*

| Study | Anxiety | Depression | Health, Well-being & Quality of Life | Caring |
| --- | --- | --- | --- | --- |
| Baker et al., 2018 |  | Patient Health Questionnaire-9 for Depression (PHQ-9) |  | Quality of the Caregiver–Patient Relationship (QCPR); Positive Aspects of Caregiving Questionnaire (PACQ) |
| Baker et al., 2012 | Geriatric Anxiety Inventory | Geriatric Depression Scale (15-item version) |  | Positive Aspects of Caregiving Questionnaire (PACQ); Mutual Communal Behaviours Scale [MCBS] |
| Brotons & Marti, 2003 | The State-Trait Anxiety Inventory (STAI) - (S-STAI*) | Beck Depression Questionnaire |  | Caregiver Burden Questionnaire (Zarit, Reever, &: Bach-Peterson, 1980) |
| Camic et al., 2013 |  |  | WHOQoL-BREF; DASS Mood Scale |  |
| Clair & Ebberts, 1997 |  | The Hamilton Rating Scale for Depression | The Positive and Negative Affect Scale (PANAS); Self-reported health | Montgomery and Borgatta Burden Scale; Boundary Ambiguity Scale for Caregivers of Patients with Dementia; Satisfaction with visits rating* |
| Clair et al., 1993 |  |  | UCLA Loneliness Scale; The Rosenberg Self-esteem Scale |  |
| Clark et al., 2020 |  | Patient Health Questionnaire-9 for Depression (PHQ-9) | Assessment of Quality of Life–8 Dimensions (AQoL-8D) | Quality of the Caregiver–Patient Relationship (QCPR); Zarit Burden Interview (ZBI) |
| Davidson & Almeida, 2014 |  |  | Bipolar five-point scales assessing: Tired – Energised, Negative Mood – Positive Mood*, Stressed – Relaxed, Unfocussed – Focused |  |
| García-Valverde et al., 2020 | The State-Trait Anxiety Inventory (STAI)- (S-STAI*) | Beck Depression Inventory Second Edition (BDI – II)* | The Rosenberg Self-esteem Scale*; Spanish version of the Short-Form Health Survey (SF-36v2) – Mental Component Summary*; Mental Health Dimension* |  |
| Hanser et al., 2011 |  |  | Visual Analog Scale (VAS): relaxation, comfort and happiness* | Caregiver burden was measured by the 5-item Caregiving Satisfaction Scale |
| Holden et al., 2019 |  |  |  | Zarit Burden Interview (ZBI); Revised Scale for Caregiving Self-Efficacy [RSCSE] |
| Melhuish et al., 2019 |  |  |  | Mental Health Foundation Carer’s Checklist |
| Mittelman & Papayannopoulou, 2018 |  | Geriatric Depression Scale (15-item version) | The Rosenberg Self-esteem Scale*; MOS Social Support Survey; SF-8 measure of Health Related Quality of Life | Communication subscale of the Family Assessment Measure |
| Raglio et al., 2016 | Hamilton Anxiety Rating Scale* | Beck Depression Inventory |  | Zarit Burden Interview (ZBI)* |
| Särkämö et al., 2014 |  |  | 12-item versions of the General Health Questionnaire (GHQ) | Zarit Burden Interview (ZBI)* |
| Tamplin et al., 2018 |  | Patient Health Questionnaire-9 for Depression (PHQ-9) | Satisfaction with Life Scale (SWLS); Flourishing Scale | 14-item Quality of Carer Patient Relationship (QCPR) scale; Positive Aspects of Caregiving Questionnaire (PACQ) |
| Zeilig et al., 2019 |  |  | Canterbury Well-Being scale (CWS) |  |

* = statistical significance
