# Supplementary material for: Integrative review of singing and music interventions for family carers of people living with dementia
Source: Health Promot Int. 2022 Apr 13;37(Suppl 1):i49–61. doi: 10.1093/heapro/daac024 (PMC9162174; doi:10.1093/heapro/daac024)
Supplement: daac024_Supplementary_Data [file daac024_supplementary_data.zip › daac024-suppl_data/Supplementary Material E.docx]

**Supplementary Material E: Integrative Review Matrix**

*Integrative Review Matrix*

| Author, Publication Year, Title | Country, Setting | Aims | Research Design | Sample | Data Collection | Summary of Key Findings |
| --- | --- | --- | --- | --- | --- | --- |
| Baker et al., (2012). Connecting through music: A study of a spousal caregiver- directed music intervention designed to prolong fulfilling relationships in couples where one person has dementia. | Australia, Home | To gain spousal carers’ perspectives of the effects of the active music therapy intervention on the spousal carer’s and spouse with dementia’s health and well-being | Mixed Methods, Quasi-experimental, No control | n=5 spousal dyads  Gender: 2 female and 3 male  Ages: 81, 81, 61, 74, 59  Ethnicity: not reported | Quantitative: pre-post-measures: Geriatric Anxiety Inventory); Geriatric Depression Scale (15-item version); Positive Aspects of Caregiving Questionnaire (PACQ); Mutual Communal Behaviours Scale [MCBS]  Qualitative: semi-structured interviews post-intervention and participants’ journal entries post-session | Null findings for quantitative measures attributed to small sample size, and low anxiety and depression scores and high quality of spousal relationship at baseline.  Qualitative findings suggest that the intervention improved mood, reduced stress, and enhanced the spousal relationship by strengthening reciprocity and giving the opportunity for shared, intimate experiences. Past experiences together involving music may contribute to effectiveness and suitability, and help to access the relationship pre-dementia. |
| Baker et al., (2018). A Group Therapeutic Songwriting Intervention for Family Caregivers of People Living with Dementia: A Feasibility Study with Thematic Analysis | Australia, Community | Determine participation and retention rates  Provide estimates on the effect of the intervention on levels of depression and the quality of the FC-PLWD relationship  Assess the acceptability and impact of treatment intervention as perceived by FC  Construct a theory of the processes activated | Mixed-methods, Quai-experimental, Self-selecting control | n=14 FC (intervention group n=8; control group n=6)  Gender: 9 female and 5 male  Age: M=72.29; SD=6.72  Ethnicity: Predominantly English-speaking backgrounds (Australia, Europe)  Relationship to PLWD: 11 spouses, 2 adult children, 1 sibling | Quantitative: pre-post-measures: Patient Health Questionnaire-9 for Depression (PHQ-9); Quality of the Caregiver–Patient Relationship (QCPR); Positive Aspects of Caregiving Questionnaire (PACQ)  Qualitative: 2 focus groups and 1 individual phone interview post-intervention | Qualitative findings suggest songwriting intervention may increase social connection and reduce loneliness; offer peer support and empathy; and foster inner strength and personal growth. It was deemed positive and worthwhile, and distinct from other support groups. It facilitated expression of the emotional journey and whole carer journey, and creativity and the presence of an end-product were valued. It was seen to enhance coping.  Qualitative findings suggest that coping may be a more relevant construct to measure than caregiver-patient relationship quality or caregivers’ perception of caregiving.  An observed pre-post effect size for the PHQ-9 in the experimental group (d = 0.64) and control group (d = −0.33) suggests the measure is sensitive to change over a short period of time in this population and has the potential to detect significant change in a larger controlled trial.  Participation and retention rates were high suggesting the intervention was acceptable. |
| Baker & Yeates (2018). Carers’ experiences of group therapeutic songwriting: An interpretive phenomenological analysis | Australia, Community | To investigate what FC of PLWD experience during group therapeutic songwriting sessions | Qualitative | n=4 FC  Gender: 2 female, 2 male  Age not reported  Ethnicity not reported  Relationship to PLWD: 2 spouses, 1 daughter, 1 son | Qualitative: Focus group and individual semi-structured interviews | Findings highlight that carers’ experiences of the programme went beyond their expectations. They found the collaborative component of co-creating the songs meaningful, and subsequently, the song held meaning for the group. FCs experienced the songwriting process as empowering. They felt like they had a voice and that it was being heard by genuinely attentive listeners. They learned about themselves, each other, and the carer journey through the process of songwriting.  Empathetic connection with other FCs through co-creativity encouraged emotional expression.  GTS was identified as being distinct from other support groups. |
| Brotons & Marti (2003). Music therapy with Alzheimer's patients and their family caregivers: A pilot project | Spain, Community | For the caregivers, the objectives were: (a) to offer a space to share experiences and life events, (b) to acknowledge and accept personal losses, and (c) to learn self-care strategies. For patients and their caregivers, the objectives addressed were: a) to enhance participation and cooperation in group tasks, (b) to foster self-esteem by experiencing success, and (c) to facilitate expression and communication through music experiences. Additional objectives are specific to PLWD and staff. | Quantitative, Quasi-experimental, No control | n=14 spousal dyads  Gender: 9 female, 5 male  Age: M=73.1, SD=6.1  Ethnicity not reported | Quantitative: Pre-post1-post2 measures: The State-Trait Anxiety Inventory (STAI); Beck Depression Questionnaire; Caregiver Burden Questionnaire (Zarit, Reever, &: Bach-Peterson, 1980); Satisfaction questionnaire. Time points = before start of intervention, 2-days before end of intervention, 2 months after intervention | The findings of the Satisfaction Questionnaire indicate that 100% of participants found the intervention relaxing and 66.7% found that it allowed them to share and express feelings that they had not been able to express before.  The results of the STAI-S yielded significant differences (X2=9.19, p=.01) suggesting that the participants were less anxious at the end of the project.  No significant differences were found in measures of depression or burden. However, scores decreased across the intervention in both cases and were lowest at post1. |
| Camic et al. (2013). Does a 'Singing Together Group' improve the quality of life of people with a dementia and their carers? A pilot evaluation study | England, Community | The present study seeks to determine if participation in a community singing group had a positive impact on both people living with dementia and their carers by increasing wellbeing, improving day-to-day functioning and reducing social exclusion. | Mixed methods, Quasi-experimental, No control | n=10 dyads  Gender: not reported  Age: not reported  Ethnicity: not reported  Relationship to PLWD: 9 spouses, 1 daughter | Quantitative: pre- post- and 10 week follow-up-measures: WHOQoL-BREF; DASS Mood Scale; observational scale to measures levels of engagement and participation; post-session anonymous evaluation cards.  Qualitative: Semi-structured interviews conducted at pre-post-and 10 week follow-up; FC diary of weekly singing and music listening activities engaged in by the dyad | Null findings for WHOQoL-BREF may be attributable to the small sample size and high level of quality of life. The fact that well-being was maintained even though PLWD was slowly deteriorating may suggest that it prevented loss of quality of life.  Very high satisfaction ratings were found.  Qualitative data suggested that the intervention improved the well-being of all participants. The findings indicated that the intervention fostered feelings of social inclusion and belonging. It was found to be relaxing, enjoyable and served as a catalyst for other musical activities that FCs and PLWD could share and enjoy together. The challenge of the musical content, and opportunity for new learning it offered, were also valued.  Feedback on practicalities and structure were positive and recommendations made. |
| Clair (2002). The effects of music therapy on engagement in family caregiver and care receiver couples with dementia | USA, Home and Residential | To explore whether music applications tailored to the preferences of the caregivers and their care recipients increase mutual engagement over time. To investigate whether mutual engagement carry over from a series of music visits to a visit without music and whether a caregiver with a non-musical background learn to implement the music application. | Mixed Methods | n=8 dyads  Gender: 5 female, 3 male  Age: M=75.25, age range=67-82 years  Ethnicity: Not specified  Relationship to PLWD: 3 husbands, 4 wives and 1 female friend | Quantitative: video recordings of sessions to record engagement data. For all couples, these behaviours included interactions between the caregiver and the care receiver, and were operationally defined as physical touch, conversation (whether or not it was verbally discernible), looking at one another, singing, vocalizing, or moving or dancing to music.  Qualitative: Informal oral questions about their experience and further engagement of protocol | All FCs were comfortable and confident with music protocol by the end of the intervention and planned to continue to use it in the future to continue mutual engagement with their care recipients. They also said that they liked the music participation very much, and they thought it added quality to the time they had with their care receivers.  Changes in engagement frequency over a series of five sessions was highly statistically significant (F=4.57, p=.006). The authors conclude that music therapy applications are effective in increasing mutual engagement in caregiving and care receiving couples with dementia, and that FCs can effectively facilitate the engagement using music. Furthermore, once the engagement is established, it carries over into visitation without music. |
| Clair & Ebberts (1997). The effects of music therapy on interactions between family caregivers and their care receivers with late stage dementia | USA, Residential | To investigate whether music therapy has an effect on carers’ perception of their feelings of (a) depression, (b) burden, (c) positive and negative affect, (d) self-reported health, and (c) satisfaction with care receivers’ visits. To explore whether music therapy has an effect on the frequency of carers’ and care receivers’ engagement with one another as indicated by initiated physical contact, or touch, and response to physical contact, or touch. To investigate whether music therapy has an effect on the frequency of carers’ and care receivers’ participation in meaningful activities including (a) conversation, (b) singing, (c) drumming, and (d) dancing? | Quantitative, Quasi-experimental, No control | n=12 dyads  Gender: not reported  Age: note specified  Ethnicity: not reported  Relationship with PLWD: not reported | Pre-post-measures: The Hamilton Rating Scale for Depression; The Positive and Negative Affect Scale (PANAS); Self-reported health; Montgomery and Borgatta Burden Scale; Boundary Ambiguity Scale for Caregivers of Patients with Dementia; Satisfaction with visits rating  Video recordings of sessions to measure frequency of carers’ and care receivers’ engagement with one another and their engagement in meaningful activities | FCs’ engagements were higher in music applications when compared to conversation, and the greatest participation occurred during rhythm playing, followed by singing and dancing, respectively. Care receivers had the greatest participation during rhythm playing, followed by dancing and singing, respectively. FCs initiated touch more frequently than their care receivers, but care receivers were more responsive to touch than their carers.  FCs’ measures of depression, burden, positive and negative affect, and self-reported health did not change, but their increased satisfaction with visits in music therapy, as compared to visits before music therapy was statistically significant (p=.017) |
| Clair et al. (1993). The effects of a socialization and music therapy intervention on self-esteem and loneliness in spouse caregivers of those diagnosed with dementia of the Alzheimer type: A pilot study | USA, Community | The purpose of the proposed study was to determine if caregivers, who had no prior musical training, could participate successfully in music with their care recipients and if participating in a social gathering with luncheon and music therapy sessions would result in changes in their loneliness and self-esteem scores. | Mixed methods, Quasi-experimental, No control | n=4 spousal dyads  Gender: 3 female, 1 male  Age: age range from 65-76 years  Ethnicity: not reported | Quantitative: pre-post-measures: UCLA Loneliness Scale; The Rosenberg Self-esteem Scale  Qualitative: pre-post-intervention interviews  Video recording of sessions | Results were not statistically significant for loneliness or self-esteem. However, for 3 participants loneliness decreased, and for 3 participants self-esteem increased. In each case 1 stayed about then same.  P1 liked the interactions but was disappointed that her spouse was not more responsive. P1 recommended that the programme should run longer, in order for it to reach its potential. P2 described it as very stimulating and a place to meet friends, and have an enjoyable experience together. P3 found it enjoyable. P4 was very satisfied. |
| Clark et al. (2020). "It's Feasible to Write a Song": A Feasibility Study Examining Group Therapeutic Songwriting for People Living with Dementia and Their Family Caregivers | Australia, Community and Residential | The study aimed to examine the feasibility of a dyad-based group TSW program. The study also aimed to test the sensitivity and appropriateness of the primary outcome measure examining relationship quality and secondary outcome measures examining quality of life and depression and perceptions of the caregiver experience. A further aim was to analyse data from post-intervention interviews with each dyad to explore participant experiences of the group TSW program and intervention design. | Mixed methods, Quasi-experimental, No control | n=14 dyads  Gender: 9 female, 5 male FC  Age: M=67.1, SD=10.1, age range=54-92  Ethnicity: Participants were from Australia, Lebanon, Italy, Malaysia | Quantitative: pre-post-measures: Patient Health Questionnaire-9 for Depression (PHQ-9); Assessment of Quality of Life–8 Dimensions (AQoL-8D); Quality of the Caregiver–Patient Relationship (QCPR); Zarit Burden Interview (ZBI)  Qualitative: Interviews post-intervention | There were no statistically significant pre- to post- differences for quantitative measures. A large pre–post effect size was observed for positive changes in the AQoL-8D independent living subscale (d = -0.93, CI = -1.8 to 0).  Qualitative data indicated that the session design and delivery were acceptable, and that TSW was a positive shared experience with personal benefits, which supported, rather than changed, relationship quality. It was seen to have a positive impact on mood and social engagement and connection. Potential barriers to PLWD engagement were noted.  TSW was recognised as being distinct from other group experiences. |
| Clark et al. (2018). Community-dwelling people living with dementia and their family caregivers experience enhanced relationships and feelings of well-being following therapeutic group singing: A qualitative thematic analysis. Tamplin et al. (2018). Remini-Sing: A Feasibility Study of Therapeutic Group Singing to Support Relationship Quality and Wellbeing for Community-Dwelling People Living With Dementia and Their Family Caregivers | Australia, Community | The aim of this study was to determine the feasibility of delivering and measuring the effects of a therapeutic group singing and home-based music program on the primary outcome of relationship quality and secondary wellbeing outcomes for PLWD and their FCs. It sought to test the feasibility of the study protocol, establish the appropriateness of the measures for answering the research questions, and collect pilot data to determine sample size for a randomized controlled trial. It strove to collect qualitative data through interviewing participants to gather information about their experience of the choir, home music program, and quantitative research measures. | Mixed methods, Quasi-experimental, No control | n=12 dyads*  Gender: 6 female, 6 male  Age: M=73.9, age range=58-88, SD=10.1  Ethnicity: Australia (n=8), Ukraine (n=1), Not reported (n=3)  Relationship to PLWD: 8 spouses, 1 daughter, 3 not reported | Quantitative: pre-post-measures: Patient Health Questionnaire-9 for Depression (PHQ-9); Satisfaction with Life Scale (SWLS); Flourishing Scale; 14-item Quality of Carer Patient Relationship (QCPR) scale; Positive Aspects of Caregiving Questionnaire (PACQ)  Qualitative: Semi-structured interviews | High participation and retention rates indicated that the intervention was received favourably by the participants. There were no statistically significant changes on measures from pre- to post-intervention. However, favourable baseline scores on relationship quality and well-being measures were sustained over the 20-week intervention. The testing of these measures for feasibility also revealed that some were too difficult for PLWD and thus yielded questionable results, some were potentially less relevant, and that there were likely floor and ceiling effects on several of the measures utilized.  Affinity with others who had similar life experiences and challenges created a sense of mutual understanding and camaraderie, which made group singing accessible without fear of judgment and social stigmas. For some PLWD/FC dyads, TGS meant they could continue a lifelong passion for singing together, while others enjoyed participating in singing together for the first time. Both PLWD and FCs described personal feelings of acceptance, improved social confidence, mood, and purpose. Further, participants valued the mental stimulation that TGS provided, such as learning new skills and memory support. A model explaining relationships between themes suggests that TGS with person-centered facilitation features for PLWD/FC dyads led to affinity among group members with ripple effects, which enhanced accessibility to group singing, the formation of empathic friendships, PLWD/FC relationship quality, and personal wellbeing for both PLWD and FCs. Psychoemotional, social and cognitive benefits from TGS described by participants in this study are known to promote self-identity, healthy relationships, and quality of life. |
| Dassa et al. (2020). Towards sustainable implementation of music in daily care of people with dementia and their spouses | Israel, Home | 1 Does the home-based model for people with dementia and their spouse PCs improve the sustainability of music in daily life? If so – how? 2 What are the recommendations for further implementation of the model? | Qualitative (Case studies) | n=2 spousal dyads  Gender: 2 female  Age: 75 and 62  Ethnicity: Israel (n=2) | Qualitative: Documentation of sessions (audiorecorded; verbal and musical content)  Documentation of phone counselling sessions  Documentation of follow-up phone session; Researcher's log | Qualitative findings indicate that the intervention aided FCs’ ability to cope. Songs became an additional caring tool, a meaningful joint activity, and positively impacted the spousal relationship, encouraging reminiscence. It also allowed one participant time for herself. |
| Davidson & Almeida (2014). An exploratory study of the impact of group singing activities on lucidity, energy, focus, mood and relaxation for persons with dementia and their caregivers | Australia Community | To examine directly whether the caregivers’ own energy, mood, level of stress/relaxation and focus was improved following attendance at the group singing session and to determine whether any additional benefit in these caregiver variables was observed when attending six consecutive weekly singing sessions. | Mixed methods, Quasi-experimental, No control | n=6 dyads  Gender: not reported  Age: age range 42-86, M=69.67, SD=16.21  Ethnicity: not reported  Relationship with PLWD: Either partner or child | Quantitative: Self-assessments, indicating where they fitted on four bipolar five-point scales assessing: Tired – Energised, Negative Mood – Positive Mood, Stressed – Relaxed, Unfocussed – Focused. Stage 1 and Stage 2 (weeks 2,4,6)  Qualitative: Very short interviews post-session on feelings, mood and experience (weeks 2, 4, 6) | Stage 1: FC energy levels, mood, and focus improved from pre- to post-singing session.  Stage 2: No findings were statistically significant. This is likely attributable to the small sample size utilised.  Qualitative data indicate that participation resulted in increased energy, improved mood, and reduced stress. The fact that the intervention required concentration enabled one FC to clear their mind of everything else. The intervention was seen to be a good opportunity for social connection. A few negative comments around mood and relaxation were noted. The intervention also facilitated positive identity construction of the care recipients, allowing FCs to see them as they were. |
| Dowlen, 2018. The 'In the Moment' Musical Experiences of People with Dementia: A Multiple-Case Study Approach | England, Community | To develop an in-depth understanding of the ‘in the moment’ musical experiences of people living with dementia when engaged with Music in Mind as a creative music-making programme | Qualitative (multiple case studies) | n=4 spousal dyads (2 additional PLWD took part)  Gender: 3 female, 1 male  Age: 75, 59, 57, 70  Ethnicity: not reported | Qualitative: video-observation; video-elicitation interviews; and participant diaries. | Music-making enabled couples to become more connected, connecting both physically (through touch) and emotionally. Spouses learnt how to support their care recipients through music. In one case there was evidence of enjoyment of singing a meaningful song together and sharing it. |
| Dupuis & Pedlar (1995). Family leisure programs in institutional care settings: Buffering the stress of caregivers | Canada, Residential | An investigation of the impacts of a shared music program on the family members of institutionalized older adults with AD that is, the role that structured family leisure may play in enhancing family visits and alleviating caregiver burden. | Qualitative | n=4 dyads  Gender: 4 female  Age: not reported  Ethnicity: not reported  Relationship to PLWD: 3 daughters, 1 wife | Qualitative: Observation: Post-session comment sheets detailing family member involvement and interactions with relatives or with other family members.  Open-ended questionnaires completed immediately post-intervention  In-depth interviews conducted 6 weeks post intervention. | The data provided evidence of social support. The intervention brought family members together, reduced loneliness and enhanced coping. A support network also developed. The data also indicated that the intervention enriched the relationship between the carer and care recipient. It enabled carers to view their relative more positively and strengthened their relationship. Enhanced quality of visits and communication were noted, with the PLWD appearing to be more relaxed and responsive. The FCs valued having the chance to contribute to their parent's or spouse's well-being. Participating in the intervention together appeared to act as a coping mechanism. It helped FC to accept the disease and situation and reduced their feelings of guilt. |
| Garabedian & Kelly (2020). Haven: Sharing receptive music listening to foster connections and wellbeing for people with dementia who are nearing the end of life, and those who care for them | Scotland, Residential | Investigate the effect of playing personalised live and pre-recorded music on solo cello on PLWD and carer | Qualitative | n=10 dyads  Gender: 7 female, 3 male  Age: mid-thirties, 46, 59, 61, 63, 65, 68, 61, 66, 83  Ethnicity: not reported  Relationship to PLWD: 6 daughters, 1 daughter-in-law, 1 son, 1 nephew, 1 3rd cousin | Qualitative: video-observations, transcribed interviews and field notes | Qualitative data captured that FCs found the intervention relaxing and that they liked seeing the PLWD enjoying themselves. Several carers spoke of experiencing tension during sessions due to feeling compelled to focus on their partner’s responses. Sharing the experience of listening to music in a safe and comfortable environment (‘haven’) facilitated re-connections; generally leading to more frequent, longer visits. |
| García-Valverde et al. (2020). The influence of songwriting on quality of life of family caregivers of people with dementia: An exploratory study | Spain, Community | 1. To examine the effect of group therapeutic songwriting on the QOL of FCs of PLWD. 2. To analyse the effect size of the intervention in the reduction of FGs’ anxiety, depression and in the improvement of their self-esteem. | Quantitative | n=21 FC  Gender: 17 female, 4 male  Age: M=65, SD=13.89  Ethnicity: not reported  Relationship to PLWD: 10 spouses, 10 adult children, 1 sibling | Quantitative: Pre-post-measures: The State-Trait Anxiety Inventory (STAI); Beck Depression Inventory Second Edition (BDI – II); The Rosenberg Self-esteem Scale; Spanish version of the Short-Form Health Survey (SF-36v2) – Mental Component Summary; Mental Health Dimension | The results showed a statistically significant decrease in anxiety and depression scores, and an increase in scores of self-esteem across the intervention. Regarding QOL, post-intervention scores in the Mental Component Summary and Mental Health Dimension were significantly higher. In contrast, a small effect size was observed for difference between pre-test and post-test on the remaining subscales of QOL: General Health, Social functioning, Role Emotional and Bodily Pain. |
| Gardner (1999). Music therapy: Enhancing communication between family caregivers and their loved ones with dementia | USA, Residential | To shed light on the process which occurs when music is used within the context of a long-term relationship between the music therapist, the FC, and the patient with dementia and investigate whether it may improve quality of life of the FCs and PLWD. | Qualitative  (Case studies) | n=2 dyads  Gender: 2 female  Age: not reported  Ethnicity: not reported  Relationship to PLWD: 2 daughters | Qualitative: Interviews, observation of music sessions with family and patients (notes, logs, cassette tape-recordings, and video recordings) | Music therapy honours the loved one with dementia and empowers the FC. FCs were empowered in moments (a) when tender emotions were shared, (b) when physical touch was used to express affection, (c) when they witnessed their loved ones actively engaged in expressing themselves through music, (d) when they took an active role in choosing music and meeting their loved ones needs on a moment-to-moment basis, (e) when they were able to soothe and comfort their loved one, and (f) when memories of past experiences and events with their loved one were stimulated by association through music. Music is something that FCs can still share with their loved ones. It provides support and facilitates meaningful interactions. It can improve FC quality of life and reduces their sense of helplessness. Music therapy enabled FCs to connect meaningfully with PLWD. It also provided them with glimpses of previous level of functioning and personality. Music therapy cannot take away the loss and grief associated with caring for a loved one with dementia. |
| Hanser et al. (2011). Home-based music strategies with individuals who have dementia and their family caregivers | USA, Home | One aim was to investigate whether caregivers who learn and guide the programme reduce their own distress and improve satisfaction with caregiving | Mixed methods, Quasi-experimental, No control | n=8 dyads  Gender: 5 female, 3 male  Age: 2 less than 65, 2 65-75, 2 76-85, 2 above 85  Ethnicity: all white, non-hispanic  Relationship to PLWD: 6 spouses, 2 daughters | Quantitative: Psychological state was measured by self-report on a Visual Analog Scale (VAS). FCs rated their own relaxation, comfort and happiness throughout baseline and after music sessions. Caregiver burden was measured by the 5-item Caregiving Satisfaction Scale at the initial session and at post-treatment exit.  Qualitative: Post-treatment interview and anecdotal reports of responses to the music, and interactions with their family member with dementia at the completion of each music listening session | FCs experienced enhanced relaxation, increase in comfort level, and happiness. These results were statistically significant. Overall, FCs experienced a greater benefit than care recipients in all three areas by an average of 1.37 points.  FCs enjoyed reminiscence, making music together and the activities. Challenges came with the responsibility of leading the music sessions themselves. Four FCs had an aversion to the verbal instructions and imagery, while two were opposed to playing instruments along with the musical selections. |
| Hanser & Clair (1995). Retrieving the losses of Alzheimer's disease for patients and caregivers with the aid of music | USA, Residential | To facilitate communication between colleagues and to test music therapy approaches across facilities and geographic areas, the authors developed individual clinical practice protocols in the hope that these would serve as replicable research models, if shown effective in a single setting. This included a protocol for music therapy for patients in early disease stages of dementia and their family carers | Qualitative (Clinical Case Study) | n=2 FC (1 PLWD)  Gender: 2 female  Age: 32, not reported  Ethnicity: not reported  Relationship to PLWD: wife and daughter | Facilitators observations and post-intervention interview | FCs developed greater self-esteem through developing their own abilities, and being able to see PLWD performing so positively and creatively. They also found the sessions relaxing. They described how they gained a new sense of their husband and father, learned new skills and shared experiences which were some of the richest in their lives. Reminiscence created meaningful experiences. |
| Harris & Caporella (2014). An intergenerational choir formed to lessen Alzheimer’s disease stigma in college students and decrease the social isolation of people with Alzheimer’s disease and their family members: A pilot study | USA, Community | The overarching goals of the choir were to reduce AD stigma and lessen social isolation. Among other aims, it sought to investigate whether social isolation could be lessened in people with AD and their family members who participated in the intergenerational choir | Qualitative | n=7 FC (n=6 PLWD, n=13 college students)  Gender: 4 female, 3 male  Age: M=72.3, age range=32-77  Ethnicity: 6 Caucasian, 1 African American  Relationship to PLWD: 5 spouses, 1 son, 1 grand daughter | Qualitative: Focus group (week 6) and observations by choir director and researcher | Participating in the intergenerational choir was found to reduce social isolation and loneliness and enabled friendships to develop. Discussing their shared experiences about AD stigma brought the dyads closer together, increasing social cohesion and lessening their feelings of isolation. The experience was found to be both beneficial and fun. |
| Harris & Caporella (2019). Making a university community more dementia friendly through participation in an intergenerational choir | USA, Community | Among other aims, this study sought to investigate whether meaningful social connections between members of an intergenerational choir of students, FC and PLWD be developed that would reach across the barriers of age, disabilities, and abilities. It also sought to investigate whether the findings be replicated across different cohorts. | Qualitative | Cohort I (n=7) Cohort 2 (n=5) Cohort 3 (n=4) Cohort 4 (n=5) Total (n=21)  Gender: 14 female, 7 male  Age: M=65  Ethnicity: 14 white, 7 black  Relationship to PLWD: 15 spouses, 3 daughters, 1 son, 2 grandchildren | Qualitative: Focus group (week 6/7 each year) and observations by choir director and researcher | The FCs experienced increased social interaction and connection, empathy, feelings of acceptance and reduced loneliness. They also developed friendships. They reported a positive experience interacting with students and felt part of a community. |
| Holden et al. (2019). Feasibility of Home-Based Neurologic Music Therapy for Behavioural and Psychological Symptoms of Dementia: A Pilot Study | USA, Home | The primary goal of this pilot study was to determine if home-based NMT was feasible for PWD and their caregivers. As exploratory outcomes, caregiver distress and self-efficacy was also evaluated. | Quantitative, Quasi-experimental, No control | n=18 dyads  Gender: not reported  Age: not reported  Ethnicity: not reported  Relationship to PLWD: not reported | Quantitative: Pre-post-measures: Zarit Burden Interview [ZBI]; Revised Scale for Caregiving Self-Efficacy [RSCSE] | No statistically significant results were found for the outcome measures.  Initiating NMT too late in the course of dementia, when behavioural symptoms are already present, may be impractical for people with dementia and increase FC stress, even when provided within the home. |
| Klein & Silverman (2012). With Love From Me to Me: Using Songwriting to Teach Coping Skills to Caregivers of Those With Alzheimer's and Other Dementias | USA, Community | The purpose of this study was to describe caregivers’ responses in a pilot intervention using songwriting to teach coping skills. | Quantitative, comparison study with psychoeducational intervention | n=14 (n=7 psychoeducational intervention; n=7 experimental music therapy songwriting group)  Gender: not reported  Age: age range 19-75  Ethnicity: not reported  Relationship to PLWD not reported | Quantitative: Questionnaire with open questions | The Linguistic Inquiry and Word Count calculated similar scores for each dimension between the psychoeducational discussion and music therapy conditions. There were nearly twice as many social words in the psychoeducational discussion condition and more words in the positive emotions dimension for the music therapy condition responses.  Themes found included: distraction from stress; reiteration of subject matter; fun; group cohesiveness; therapeutic insight; appreciation; and comment on presentation. The music therapy condition had more responses in the “fun,” “appreciation,” and “comments on presentation” than did the psychoeducational condition. FCs expressed happiness at observing their family members being able to offer their talents and contribute to the sessions. |
| Lee et al. (2020). Promoting wellbeing among people with early-stage dementia and their family carers through community-based group singing: a phenomenological study | Ireland, Community | To investigate the impact of a community-based group singing intervention on the well-being of people living with early-stage dementia and their family carers | Qualitative | n=4 FC (n=3 PLWD) Gender: 3 female, 1 male Age: 1 30-39; 1 60-69, 2 70-79 Ethnicity: not reported Relationship to PLWD: 3 spouses, 1 daughter | Qualitative: Semi-structured interviews post-intervention | Interpretative phenomenological analysis revealed four superordinate themes: social connection; happiness and rejuvenation; reconnection with the self; and supporting the carer–cared-for relationship.  There was evidence of multidimensional enhancement of well-being.  Musical activities were conducive to active engagement, and the small group size and similar cognitive capacities encouraged participation. The participants were united by their common interest in music, which provided them with a sense of familiarity and confidence in a new social situation.  The intervention was recognised as being a good opportunity to spend meaningful one-to-one time with their family member, where their roles were incidental and could be forgotten. |
| Macgregor (2016).  Music therapy: A bridge to communication for familial caregivers of persons with dementia | USA, Home | Among other aims, this study investigated how the use of familiar music may elicit emotional reciprocity and meaningful communication between a person with dementia and their familial caregiver | Qualitative  (Collective Case Study Design) | n=4 dyads  Gender: 3 female, 1 male  Age: age-range 47-85  Ethnicity: not reported  Relationship to PLWD: 2 spouses, 1 best friend, 1 sibling | Qualitative: The data collected during this study consists of audio recordings of pre-and post-intervention interviews, transcripts (verbatim) of those recorded interviews, FC respondents' journal logs, and the researcher's observational field notes and journal log | The dyads experienced emotional reciprocity though music. This renewed the FCs’ expectations and gave them a new way of being together: communication through movement and music. The FCs were delighted at the increase in responsiveness and engagement of the PLWD. The experience brought them closer together and gave the FCs an insight into aspects of PLWD's personality through the music. By the conclusion of the study the FCs had come around to a slightly different perspective of meaningful communication and appeared to value non-verbal communication to a higher degree. |
| Melhuish et al. (2019). Mindsong, music therapy and dementia care: collaborative working to support people with dementia and family carers at home | England, UK, Home | To evaluate the effects of providing music therapy at home for couples facing the challenges of dementia and to develop a robust delivery model through forging new working relationships with local organisations. | Mixed methods, Quasi-experimental, No control | n=8 spousal dyads  Gender: 5 female, 3 male  Age: 72, 70, 73, 77, 84, 83, 73 (one missing as withdrew from study)  Ethnicity: not reported | Quantitative: pre- post-14 days, 3 month follow-up measures: Mental Health Foundation Carer’s Checklist. It records dementia driven behaviours and how stressful these are for the carer. It also asks the carer to rate the burden of caring in terms of social, financial, physical and emotional strain.  Qualitative: Mindsong Questionnaire to obtain carer feedback on the impact of music therapy for both themselves and their relative was completed at 14 days post and 3 month follow-up.  MTs clinical notes and case reports. | For three couples carer stress was significantly lower post intervention, with reductions in scores of 47%, 46% and 32%. One of these carers was ‘despairing and angry’ when first assessed but afterwards was ‘in control again’, while another was less stressed by the need to always to be present and ‘quite happy to devote my time to [name]’. Recorded stress was higher post intervention for two FCs and not significantly changed for the remaining two FCs.  The intervention played a role in counteracting social isolation for FCs. Helping FCs to extend and strengthen formal and informal support networks contributed to increasing their resilience and feelings of empowerment. The FCs valued being able to do something with the PLWD and the opportunity to share humour and affection and enjoy reminiscence together. It made them feel more connected to their spouse. |
| Mittelman & Papayannopoulou (2018).  The Unforgettables: a chorus for people with dementia with their family members and friends | USA, Community | The goals of the pilot study were two-fold: (1) to fine-tune the intervention so that it would be enjoyable and appropriate to both the person with dementia and the caregiver, neither too difficult for the former nor to demeaning for the latter; (2) to obtain initial qualitative and quantitative data on the potential benefits of the intervention. | Mixed methods, Quasi-experimental, No control | n=11 dyads  Gender: 6 female, 5 male  Age: M=71.7, SD=8.3  Ethnicity: Caucasian, 10 non-hispanic, 1 hispanic  Relationship with PLWD: 9 spouses/partners, 1 adult child, 1 close friend | Quantitative***:*** Pre-and post-measures: demographic questions, the Communication subscale of the Family Assessment Measure; Rosenberg Self-esteem Scale; MOS Social Support Survey; the SF-8 measure of Health Related Quality of Life and the Geriatric Depression Scale (15-item version.  Qualitative: Take-home questionnaires that included structured and open ended questions were given to participants after the last rehearsal. Two focus groups held one week after the concert for in-depth exploration of participants’ reactions to the program (informal thematic analysis conducted) | For FCs, self-esteem was the only outcome to improve significantly based on the t-test, but showed a medium large effect size (t =2.15; p = 0.060, d = 0.68). While change in SF-8 (t = 1.42, NS, d = 0.45) and social support (t = 1.32, NS, d = 0.42) were not significant, both outcomes produced a medium effect size. FC depression was very low before the intervention, and did not change with the intervention.  Participation in the intervention had a lasting benefit on FCs moods. Increased self-care, sense of community, value of the social support of the group, interacting with new people, exchanging ideas, having ‘an enjoyable and happy time,’ and sharing an experience with other FCs and people with AD were among the benefits cited. The participants enjoyed the challenge of learning new songs and singing techniques, and the pleasure of the activity itself. They also enjoyed seeing its positive impact on the PLWD. Conductors and staff were seen to have created a nurturing and stimulating environment. |
| Osman et al. (2016). ‘Singing for the Brain’: A qualitative study exploring the health and well-being benefits of singing for people with dementia and their carers | England, Community | To explore the experiences of people with dementia and their carers attending a group singing activity. | Qualitative | n=10 dyads  Gender: 8 female, 2 male  Age: not reported  Ethnicity: White British  Relationship to PLWD: 5 wives, 2 husbands, 3 daughters | Qualitative: Semi-structured interviews. | Social inclusiveness, support, belonging, enduring positive effect on their sense of well-being and capacity to provide care were cited as benefits of participation. Participating also had a positive impact on their relationship with the PLWD as it added a new dimension, stimulated conversation, stirred memories and reminiscence, and helped them to cope with diagnosis. The FCs valued that this meaningful interaction not focused on an aspect of clinical care. |
| Raglio et al. (2016). Active music therapy for persons with dementia and their family caregivers | Italy, Home | One of the aims of this study was to investigate possible effects of AMT in improving psychological and stress conditions in FC. | Quantitative, Quasi-experimental, No control | n=4 spousal dyads  Gender: 3 female, 1 male  Age: not reported  Ethnicity: not reported | Quantitative: A psychological assessment was made using the Burden Interview, the Hamilton Anxiety Rating Scale, and the Beck Depression Inventory to evaluate the stress level, anxiety, and depression in FC. Clinical evaluations were made at T0, T1, and T2 (respectively before and after the treatment and 1 month after the end of AMT sessions). | Significant results were obtained on FCs in anxiety and burden reduction in which all subjects showed a remarkable improvement at T1 and sometimes also at follow-up. Also in depression symptoms, the most part of FCs slightly improved. |
| Särkämö et al. (2013). Role of musical leisure activities in dementia care: Applicability and benefits perceived by caregivers; Särkämö et al. (2014). Cognitive, emotional, and social benefits of regular musical activities in early dementia: Randomized controlled study | Finland, Community and Residential | This study aimed to determine the efficacy of a novel music intervention based on coaching the caregivers of PWDs to use either singing or music listening regularly as a part of everyday care. | Randomised Controlled Trial (RCT) (Singing Group, Music Listening Group, Control Group) | n=59 dyads (n=30 nurse-PLWD dyads)  Gender: 41 Female, 18 male  Age: not reported  Ethnicity: not reported  Relationship to PLWD: 29 spouses, 19 adult children, 11 siblings | Quantitative: pre-follow-up1 (3 months from baseline), follow-up 2 (9 months from baseline) measures: 12-item versions of the General Health Questionnaire (GHQ); Zarit Burden Interview (ZBI). The GHQ contains questions related to anxiety and depression, social dysfunction, and loss of confidence, and the ZBI contains questions about the experienced strain and burden as a caregiver.  Qualitative: Short semi-structured telephone interviews were conducted at Follow-up 2. During this the FCs were asked to answer a number of questions relating to their experiences on 10-point Likert scales (Quant) | A significant long-term specific effect was observed for the ZBI scores, Time × Group F(2, 44) = 4.0, p = .026, which decreased (indicating reduced burden) more in the SG than in both MLG (p = .029) and CG (p = .069) from baseline to Follow-up 2. The same effect was observed also for the GHQ, but it failed to reach statistical significance (Time × Group F(2, 44) = 2.5, p = .095; ANCOVA group effect F(2, 43) = 1.8, p = .174). Thus, it appears that singing, in particular, was beneficial for the emotional well-being of the FCs.  Continuing the musical activities regularly at home seems to be linked to better mood, QOL, and memory of the PLWD, promoting the well-being of FCs in mild/moderate dementia. |
| Shibazaki & Marshall (2017).  Exploring the impact of music concerts in promoting well-being in dementia care | England and Japan, Residential | The aim of this study was to investigate what ways can live music concerts influence clients living with dementia, their caregivers and their family members | Qualitative | n=13 FC (UK n=8; Japan n=5)  Gender = not reported  Age = not reported  Ethnicity = not reported  Relationship to PLWD = not reported | Qualitative: Interviews | The musical performances provided some of the most lasting and significant memories and experiences for the families of clients who attended. According to comments made by families, the music stimulated new memories, extended conversations, created increased quality visitor experiences and partially restored lost or diminished aspects of personality. Levels of guilt decreased as relatives were seen to be experiencing something they could not have been given at home. They also gave the FCs an insight into PLWD’s health and well-being. Only 2 instances where the concert was viewed as an inconvenience were reported. |
| Unadkat et al. (2017). Understanding the experience of group singing for couples where one partner has a diagnosis of dementia | UK (England and Wales) | This study sought to investigate how group singing can impact the relationship between the person with dementia and their spouse and discover what the key factors involved in this experience for the couple are | Qualitative | n=17 spousal dyads  Gender: 9 female, 8 male  Age: M=70.3, range 61-89  Ethnicity: not reported | Qualitative: Interviews | Group singing was found to be uplifting, therapeutic and to positively impact mood. The participants valued belonging to a social group, sharing an experience, and forming a group identity. The capacity of music to facilitate shared experiences and bring people together was identified. Benefits were also observed for PLWD-FC relationship and they valued having the opportunity to engage in something together creating new experiences rather than just looking back and reminiscing. The excitement around the performance element was clear and the participants spoke about the anticipation of looking forward to this element which often is not present in other groups. Effective group facilitation was deemed essential. Singing was also described as an activity easily carried out by both partners at home together. Encouraging participation, person-centeredness, and equality were identified as part of the role of an effective group facilitator. Effective facilitation enabled a ‘release’ from caring responsibilities and increased awareness of the PLWD’s identity outside the diagnosis. The release of carer burden appeared to link together to create a benefit of changing roles and equal footing. If the PLWD was the better singer the dyad might actually experience role reversal. It inspired couples to join other activities or groups. There was a sense of excitement, a desire to look forward and an appreciation of learning a new skill. |
| Zeilig et al. (2019). Co-creativity, well-being and agency: A case study analysis of a co-creative arts group for people with dementia | England (UK), Community | This study investigates how co-creativity can affect well-being from the perspectives of people with dementia and their carers; and explores how well-being and agency might be usefully reconsidered. | Mixed Methods (Primarily qualitative) | n=3 FC (the group comprised 3 musicians, 2 dancers, 2 researchers, 5 PLWD and 3 FC)  Gender: Not reported  Age: Not reported  Ethnicity: Not reported  Relationship with PLWD: 3 partners | Qualitative: Dialogic interviews, video data, and field notes  Quantitative: Canterbury Well-Being scale (CWS) (Interest, Confidence, Optimism, Well, Happiness) | Composite CWS score for FCs: there was no data to calculate average difference before and after all sessions combined since there was no data available for the FCs before and after session one. When looking at the three time points separately, although there was an increase in CWS scores at the three time points where data was available, this was not a statistically significant difference for the FCs.  The intervention was based around improvisation and had a consequent lack of preconceptions or expectations. There was no predetermined sense of direction. However, while this invoked a sense of self sufficiency and freedom that are features of autonomy, and novelty, the lack of direction made some participants feel uncomfortable and vulnerable. Similarly, the lack of leadership stimulated sharing of role and spontaneity, but some participants felt that it lacked direction. The confrontation of vulnerability facilitated an exploration of challenging emotions and issues, such as anger and death. A sense of community and connection developed within the group, characterised by shared experience and mutual empathy. The art forms were accessible, and the experience was described as rejuvenating and nourishing and playful. It was also observed to improved confidence. |

N.B. In each case only the information specifically relevant to family carers is presented (e.g. studies may have had additional aims, findings etc.)

*As reported by Tamplin et al., 2018. Clark et al., 2018 reports only includes the participants that completed the intervention (n=9).
