# Supplementary material for: Integrative review of singing and music interventions for family carers of people living with dementia
Source: Health Promot Int. 2022 Apr 13;37(Suppl 1):i49–61. doi: 10.1093/heapro/daac024 (PMC9162174; doi:10.1093/heapro/daac024)
Supplement: daac024_Supplementary_Data [file daac024_supplementary_data.zip › daac024-suppl_data/Supplementary Material F.docx]

**Supplementary Material F: Integrative Review Music Interventions**

*Music Interventions Included in the Integrative Review*

|  | Type of Music Intervention | Facilitator(s) | Length of Music Intervention | Brief Description of Musical Content |
| --- | --- | --- | --- | --- |
| Baker et al., 2012 | Active music intervention programme (spousal dyad) | Family Carer (Initial instructional session with MT) | 6 weeks, 3 20-30 minute sessions per week | The intervention comprised of 3 main activities: singing familiar/preferred songs; movement to music; listening to quiet relaxing music with eyes closed |
| Baker et al., 2018 | Therapeutic songwriting (family carer groups) | Music Therapist | 6 weeks, 1 hr per week | Songwriting – co-creating and recording a song about their caring experiences |
| Baker & Yeates, 2018 | Therapeutic songwriting (family carer group) | 2 Music Therapists | 4 1hr sessions | Songwriting – co-creating a song about their caring experiences |
| Brotons & Marti, 2003 | Music Therapy (groups) | 2 Music Therapists | 12 days, 7 sessions for FC and PLWD together; 4 sessions for FC alone (and 10 sessions for PLWD alone) | The intervention included a variety of music activities: music listening, singing, instrument playing, and movement/dance, instrumental ensembles, and sing-alongs |
| Camic et al., 2013 | Group Singing (‘Singing Together Group’) (group, caring dyads) | Qualified Community Musician | 10 weeks, 90 minutes | Sessions involved vocal warm-ups and singing a mixture of well-known, new and requested songs, with some performed as rounds. Small percussion instruments were available for use. |
| Clair, 2002 | Music Therapy Applications (MT instructed FC how to use music with PLWD) (caring dyad with MT) | Music Therapist and Family Carer assumed leadership roles at different points during the intervention | 8 weeks, 40 minutes per week | Participants selected either a singing or dancing protocol. It was adapted for each dyad. The singing protocol involved the FC cuing favourite songs and one member of the dyad accompanying on a musical instrument. The dancing protocol involved the FC initiating dances with PLWD to recorded music |
| Clair & Ebberts, 1997 | Music Therapy (small groups, 2-4 caring dyads) | Music Therapist | 8 sessions, 50 minutes, twice weekly | 10 minutes of each of (a) initial conversation, (b) singing, (c) ballroom, folk or chair dancing, (d) rhythm participation using drums, (e) follow-up conversation |
| Clair et al., 1993 | Music Therapy (group, caring dyads) | 2 Music Therapists (and social worker) | 6 weeks, 1 30 minute session per week | 10 minutes of group singing, 10 minutes of hoop drum playing, and 10 minutes of ballroom dancing |
| Clark et al., 2020 | Therapeutic Group Songwriting (small groups of 2-3 caring dyads) | Music Therapist(s) | 6 weeks, 1 hr per week | The intervention was influenced by concepts of personhood, couplehood, and group process, in combination with an experience-based TSW model. Sessions included opportunities for brainstorming, reminiscence, and lyric creation, as well as music listening, familiar song singing, and instrument playing. |
| Clark et al., 2018; Tamplin et al., 2018 | Therapeutic Group Singing (group, caring dyads) | 2 Music Therapists | 20 sessions, approx. 2 hrs | The therapeutic group singing sessions were based on Kitwood’s concepts of personhood. Each session included introductions and information updates (5–10 min), vocal warm ups and exercises (15–20 min), singing familiar participant requested songs (30–45 min), learning new songs and singing skills introduced by the researchers (20–30 min), and socialization over afternoon tea (30 min). |
| Dassa et al., 2020 | Music Therapy (and bi-weekly pone counselling sessions) (spousal dyad and MT) | Music Therapist | 12 weekly sessions | Musical activities included singing, listening to music, dancing and playing percussion instruments. The MT matched her musical interventions according to the couple’s preferences and reactions at each session. |
| Davidson & Almeida, 2014 | Group Singing (group, caring dyads) | Facilitator - expert in singing and considerable experience working in the community and with older groups | Stage 1 = 1 singing session  Stage 2 = 6 singing sessions, 2 hrs long | Building and progressing vocal warm-ups, main repertoire, musical games and tea break. Followed principles outlined by Davidson and Faulkner (2010) |
| Dowlen, 2018 | Music in Mind (group, FC and PLWD) | 1 music therapist, 1 Manchester Camerata musician | 15 weeks (90 minutes – 60 music, 30 social) | Music in Mind programme. Its key principles are improvisation and creativity; choice; and co-facilitation. |
| Dupuis & Pedlar, 1995 | Reminiscence music programme (group, caring dyads) | Not specified (but designed by author) | 12 1 hr sessions, 2 per week for 6 weeks | Reminiscence music programme. Songs were chosen for their potential to evoke the discussion of past memories on a certain topic. No further detail given. |
| Garabedian & Kelly, 2020 | Individualised receptive music listening interventions (3 live, 3 recorded) (caring dyad and facilitator) | Professional cellist/certified music and health practitioner | 6 sessions – 15-70 minutes | Receptive listening to personalised music played on solo cello. |
| García-Valverde et al., 2020 | Therapeutic Songwriting (family carer groups) | Music Therapist | 12 60 minute sessions | A therapeutic model was developed, consisting of (1) an introductory phase utilising therapeutic  improvisation, therapeutic singing, and receptive methods; (2) a phase of exploration and development incorporating therapeutic singing and musical collage; (3) creation of lyrics; (4) creation of music; (5) instrumentation recording and closing |
| Gardner, 1999 | Music Therapy (caring dyad and MT) | Music Therapist | Approx. 1 year (1 dyad 10 months; 1 dyad 18 months), 30-60 minutes weekly | Sessions included singing, listening to tapes, instrument playing, movement, dancing, and discussion. Musical accompaniment was provided primarily with guitar. Instrument playing included use of small rhythm  instruments, drums, and omnichord. The content of each session was determined by individual needs. |
| Hanser et al., 2011 | Music-facilitated stress reduction program (music listening) (caring dyad) | Family Carer (trained by MT 2 hr session) | Number of sessions determined by FC. Target goal of 8-20 total sessions. | Personalised recorded music with recorded instructions was used. The FC initiated one or more of the 8 methods of engaging with the recorded music contained in the protocol with their care recipient during each session. |
| Hanser & Clair, 1995 | Music Therapy (group, family members and PLWD) | Music Therapist | 8 weeks, 1 hr per week | There were four distinct parts to each session: music listening for stress reduction; singing; improvising; and music listening with discussion. |
| Harris & Caporella, 2014 | Intergenerational Choir for college students, PLWD and FC | Choir Director | 8 weeks, 90 minute rehearsals before concert | Choir rehearsals were adapted to promote social interaction and contact between the students and PLWD and FC using 5 methods. Musical content was considered carefully in relation to the participant groups. |
| Harris & Caporella, 2019 | Intergenerational Choir for college students, PLWD and FC | Choir Director | 10 weeks, 90 minute rehearsals before concert (on four separate occasions) | Choir rehearsals were adapted to promote social interaction and contact between the students and PLWD and FCs using 5 methods. Musical content was considered carefully in relation to the participant groups. |
| Holden et al., 2019 | Neurologic Music Therapy (caring dyad and 1 MT) | 4 Music Therapists | 6 weeks, session 1 = 90 minutes, sessions 2-6 = 60 minutes | A combination of MSOT, MACT, and AMMT techniques were incorporated into the NMT protocol. The sessions began with  MSOT techniques, including both a sing-along and a play-along portion with an instrument of their choice. They then moved into higher level attention tasks utilizing MACT. Finally music and memory reminiscence was facilitated through AMMT. |
| Klein & Silverman, 2012 | Therapeutic songwriting (group) | 4th year MT student | 1 session, 45 minutes | The song ‘With Love From Me to You’ was introduced as the basis of a new composition about coping skills to be written by the group with the new title ‘With Love From Me to Me’. This was a lyric replacement intervention with guitar accompaniment. Guiding questions were posed to facilitate lyric composition. |
| Lee et al., 2020 | Singing Group (caring dyads) | Music Therapist | 6 weeks, 1 hr per week | Each singing session followed a flexible structure and included musical elements that promoted the following: (a) social connection; (b) expression and creativity in-the-moment; (c) reminiscence; (d) life-long learning and cognitive stimulation. The facilitator adopted a person-centred approach and participants played an active role, suggesting songs and music genres. Sessions began and ended the same way each week (vocal warm-ups; goodbye song ‘Hit the Road Jack’ with participants’ names) to promote familiarity. |
| Macgregor, 2016 | Music Therapy (caring dyads and MT student) | Music Therapy Student and Family Carer | 4 weeks, multiple sessions per week, FC to facilitate on at least two occasions alone) | Musical activities including receptive listening; movement to music; and active music-making using familiar music. |
| Melhuish et al., 2019 | Mindsong Music Therapy (spousal dyads) | Music Therapists | 12 weekly sessions, up to 1.5 hrs | Mindsong Music Therapy is person-centred and individualised to the care dyad and may use familiar songs, improvisation and listening to music as appropriate. Instruments may include laptop snare, maracas, tambourines, metallophone and deskbells to stimulate rhythmic and melodic responses and improvisation within the dyad is encouraged. The guitar is often used by the therapist to support singing and an iPad can be a useful resource for listening to preferred music. |
| Mittelman & Papayannopoulou, 2018 | Chorus for PLWD, FC, friends called ‘Unforgettables’ | Choral conductor, assistant conductor and music therapist | 13 2 hr rehearsals and concert | A wide range of repertoire is employed. During rehearsals, conductors teach performance and vocal techniques, including breathing, phrasing, singing in other languages, and concert preparation. They try to promote self-expression, creativity, self-esteem, a good mood, meaningful connections, sense of community, and social support. Warm ups and rhythm techniques are central. |
| Osman et al., 2016 | Group Singing (‘Singing for the Brain’) (caring dyads) | Musician | Participants had to attend a minimum of 2 Singing for the Brain sessions. | A Singing for the Brain session starts with warm-up exercises for voice and body before moving on to singing familiar songs that follow a different theme each week. Attendees are provided with song sheets including the lyrics for each song. Songs are sung in unison accompanied by the musician or in rounds with harmonies. Depending on access to equipment, attendees can use percussive instruments during the session. At the beginning and end of each session, there is an opportunity for refreshments and time to socialise. |
| Raglio et al., 2016 | Structured Active Music Therapy Intervention (spousal dyad and MT) | Music Therapist | 12 sessions, 40 minutes each, twice a week | No detail given |
| Särkämö et al., 2013; Särkämö et al., 2014 | Group-based music coaching program. 2 available interventions: singing sessions and music listening sessions (groups, familial caring dyads, prof caring dyads) | Music Teacher – singing groups; Music Therapist – music listening groups | 10 weeks, 1.5 hrs per week, carers encouraged to continue singing and listening at home during and post intervention | Singing group sessions consisted primarily of singing songs in a group accompanied by the music teacher on the piano, guitar, or kantele (Finnish zither), as well as occasionally performing physically activating vocal exercises and rhythmical movements during the singing (e.g., clapping, playing maracas). The music listening group sessions consisted primarily of listening to songs from CD and discussing about the emotions, thoughts, and memories (e.g., personal events, people, and places) that they evoked. Also visual cues (e.g., album covers) were used to stimulate reminiscence and discussion. |
| Shibazaki & Marshall, 2017 | Live Concerts | N/A | 22 1 hr concerts took place (11 England, 11 Japan) | In England, all performers were professional musicians performing as a duet. All concerts contained a similar style of repertoire, including instrumental music for listening as well as familiar songs/pieces which residents could join in with and sing, including songs from shows, music from popular films, well known traditional songs and popular items from the western classical tradition. In Japan, the repertoire similarly included songs from the time of the Second World War, classical piano pieces, and traditional, community and popular songs/pieces from the culture. |
| Unadkat et al., 2017 | 5 different types of singing groups for PLWD and FC | Not specified | Different durations | The five types of singing groups were described as: (1) 7 session singing and composing group held by art gallery and opera company in which people with dementia and their caregivers composed an opera based on art in the gallery. Ended in a performance and a follow-up reunion. (2) Ongoing group singing involving music, movement, and percussion for people with dementia and their caregivers; (3) Ongoing choir for people with dementia and their caregivers. Regular performances and a CD recording. (4) Ongoing group singing hosted by a national charity involving reminiscence singing and movement. (5) 10 session group singing and music making program for people with dementia and their caregivers. |
| Zeilig et al., 2019 | Co-creative group arts sessions ‘With All’ involving music and dance | No facilitator –co-creative group | 4 weeks, 1 hr per week | Free, unstructured creative process. The co-creative sessions were based on improvisation and a consequent lack of preconceptions or expectations. |
