# Supplementary figures and images for: Integrative review of singing and music interventions for family carers of people living with dementia
Source: Health Promot Int. 2022 Apr 13;37(Suppl 1):i49–61. doi: 10.1093/heapro/daac024 (PMC9162174; doi:10.1093/heapro/daac024)

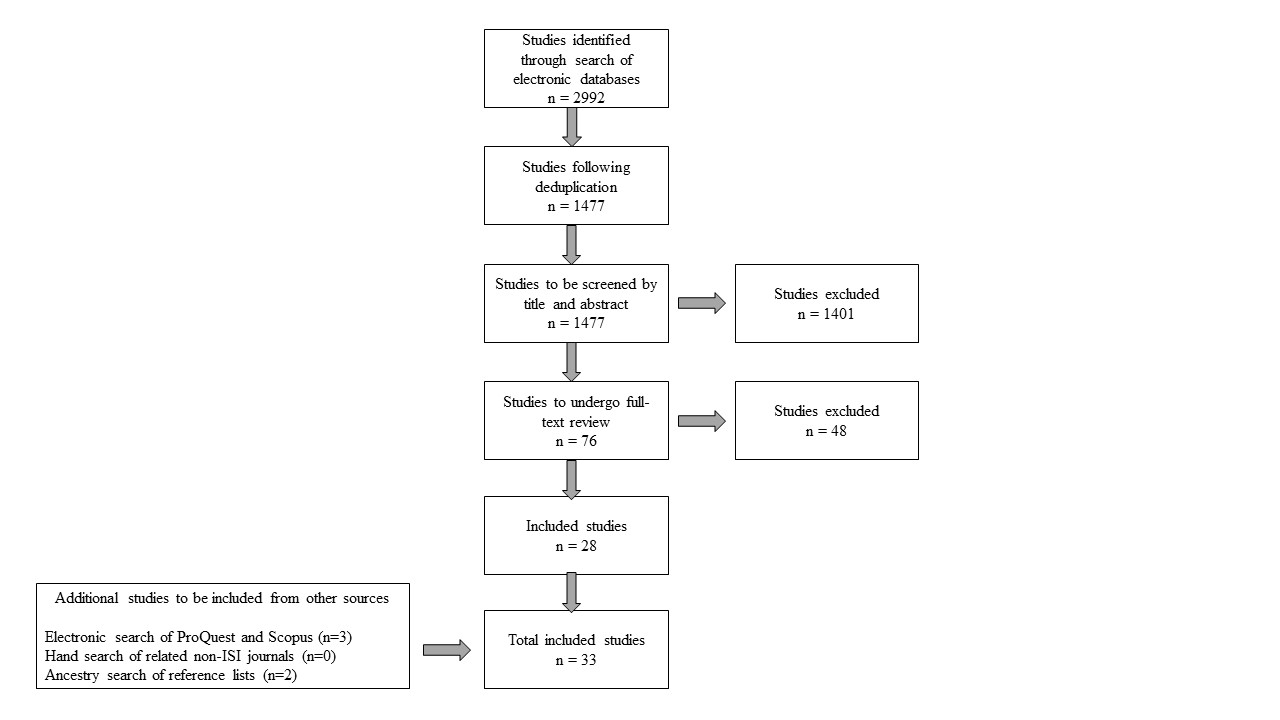

Supplement: daac024_Supplementary_Data [file daac024_supplementary_data.zip › daac024-suppl_data/Supplementary Material B Figure S1 Literature Search Results.jpg]

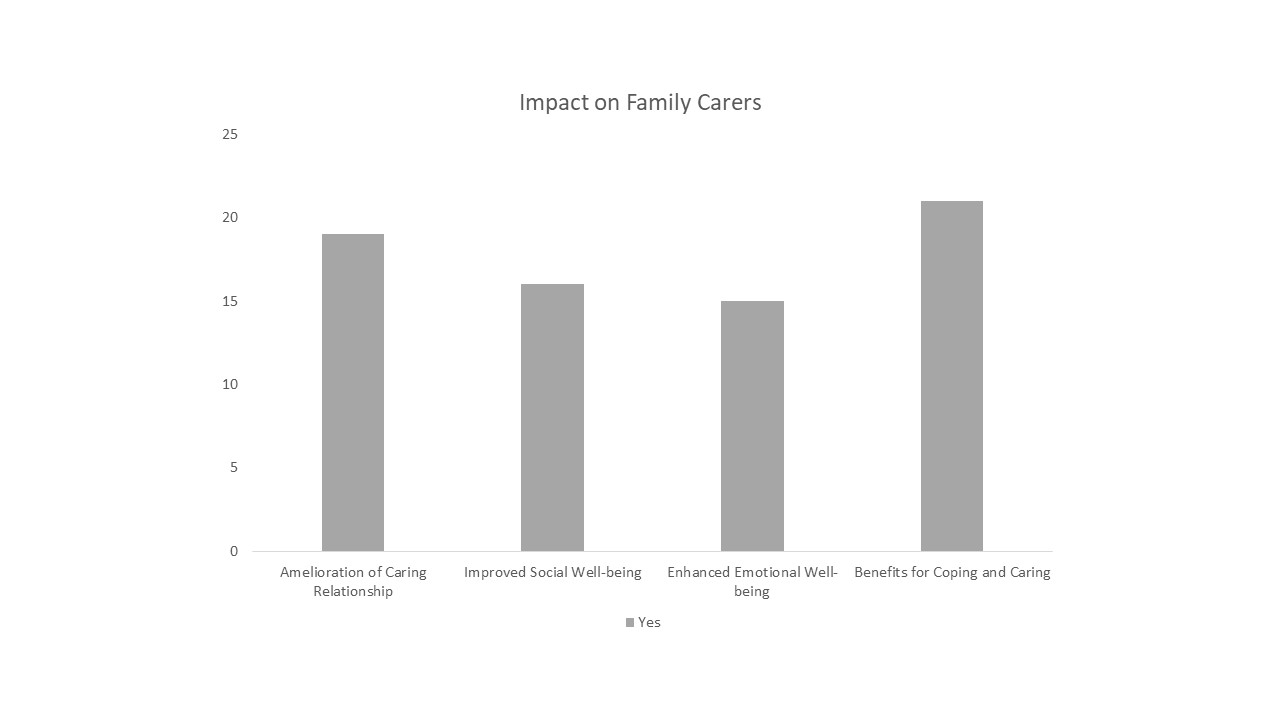

Supplement: daac024_Supplementary_Data [file daac024_supplementary_data.zip › daac024-suppl_data/Supplementary Material G Frequency of Subthemes within Overarching Themes Figure S2 Impact on Family Carers.jpg]

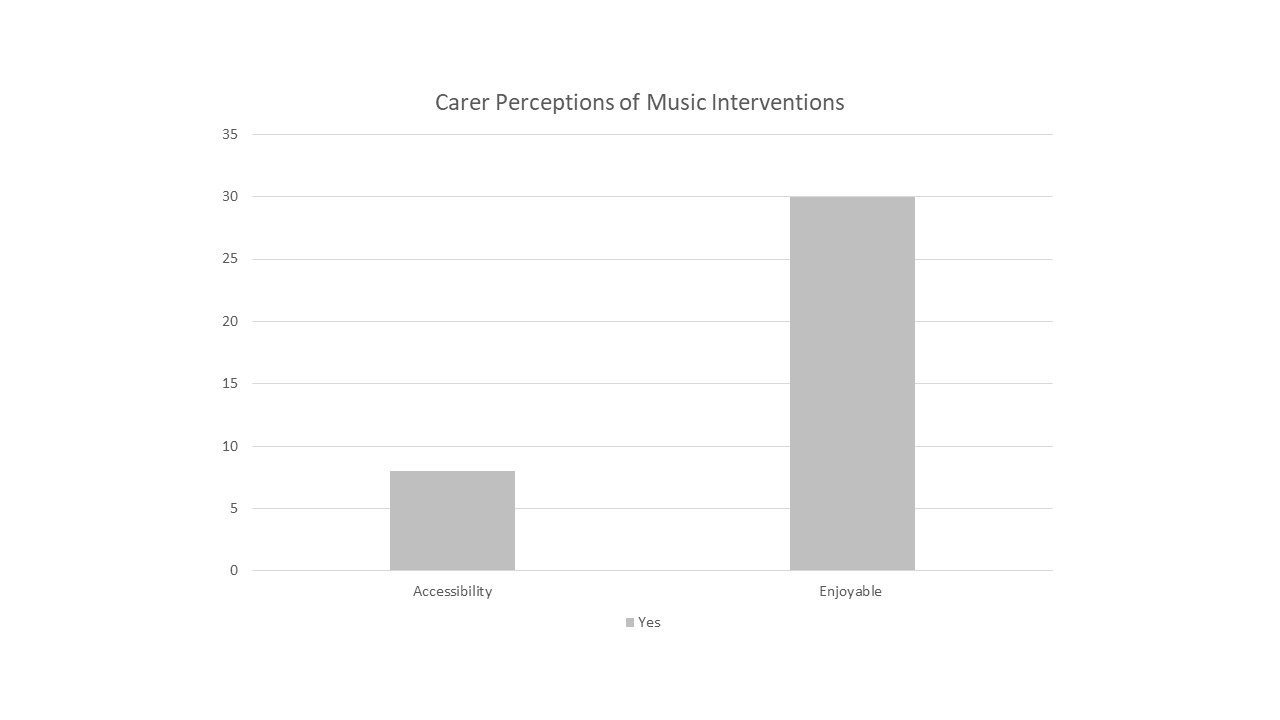

Supplement: daac024_Supplementary_Data [file daac024_supplementary_data.zip › daac024-suppl_data/Supplementary Material G Frequency of Subthemes within Overarching Themes Figure S3 Carer Perceptions of Music Interventions.jpg]
